# Practice variation in the use of tests in UK primary care: a retrospective analysis of 16 million tests performed over 3.3 million patient years in 2015/16

**DOI:** 10.1186/s12916-018-1217-1

**Published:** 2018-12-20

**Authors:** Jack W. O’Sullivan, Sarah Stevens, Jason Oke, F. D. Richard Hobbs, Chris Salisbury, Paul Little, Ben Goldacre, Clare Bankhead, Jeffrey K. Aronson, Carl Heneghan, Rafael Perera

**Affiliations:** 1 Centre for Evidence-Based Medicine, Nuffield Department of Primary Care Health Sciences, University of Oxford, Radcliffe Observatory Quarter, Oxford, OX2 6GG UK; 2 Nuffield Department of Primary Care Health Sciences, University of Oxford, Oxford, OX2 6GG UK; 3 Centre for Academic Primary Care, Department of Population Health Sciences, Bristol Medical School, University of Bristol, Bristol, BS8 2PS UK; 4 Primary Care and Population Sciences, University of Southampton, Southampton, SO17 1BJ UK

**Keywords:** Overuse, Health policy, Primary care, General practice, Test use, Imaging

## Abstract

**Background:**

The UK’s National Health Service (NHS) is currently subject to unprecedented financial strain. The identification of unnecessary healthcare resource use has been suggested to reduce spending. However, there is little very research quantifying wasteful test use, despite the £3 billion annual expenditure. Geographical variation has been suggested as one metric in which to quantify inappropriate use. We set out to identify tests ordered from UK primary care that are subject to the greatest between-practice variation in their use.

**Methods:**

We used data from 444 general practices within the Clinical Practice Research Datalink to calculate a coefficient of variation (CoV) for the ordering of 44 specific tests from UK general practices. The coefficient of variation was calculated after adjusting for differences between practice populations. We also determined the tests that had both a higher-than-average CoV and a higher-than-average rate of use.

**Results:**

In total, 16,496,218 tests were ordered for 4,078,091 patients over 3,311,050 person-years from April 1, 2015, to March 31, 2016. The tests subject to the greatest variation were drug monitoring 158% (95%CI 153 to 163%), urine microalbumin (52% (95%CI 49.9 to 53.2%)), pelvic CT (51% (95%CI 50 to 53%)) and Pap smear (49% (95%CI 48 to 51%). Seven tests were classified as high variability and high rate (clotting, vitamin D, urine albumin, prostate-specific antigen (PSA), bone profile, urine MCS and C-reactive protein (CRP)).

**Conclusions:**

There are wide variations in the use of common tests, which is unlikely to be explained by clinical indications. Since £3 billion annually are spent on tests, this represents considerable variation in the use of resources and inefficient management in the NHS. Our results can be of value to policy makers, researchers, patients and clinicians as the NHS strives towards identifying overuse and underuse of tests.

**Electronic supplementary material:**

The online version of this article (10.1186/s12916-018-1217-1) contains supplementary material, which is available to authorized users.

## Introduction

Healthcare systems around the world are struggling to remain fiscally sustainable [1–3]. With increases in spending, healthcare systems are faced with a mismatch between funding and expenditure [4, 5].

To help reduce costs, the identification of unnecessary care has become a focus of governments and healthcare funders around the world [6]. Primary care accounts for most health care (90% of all UK National Health Service (NHS) care [7], 55% in the USA [8]) and serves a gatekeeper function in the UK; tests often have knock-on consequences in both primary and secondary care. As such, the identification of wasteful resource use in primary care has implications for the entire healthcare system.

Previous research has suggested that when there is strong evidence and a professional consensus that an intervention is effective, there tends to be almost no variation in practice [9, 10]. Conversely, variation in the use of resources has been used to highlight possible overuse or underuse [10, 11].

Despite its contribution to care and expenditure [12], there is little research into variation in the use of tests by general practitioners (GPs). UK GPs are thought to spend more than £3 billion annually on tests [12]. One study has explored variation in GP test use, but it focused on a few specific tests in a relatively small population [13]. We set out to identify which tests are subject to the greatest between-practice variation in their use.

## Methods

### Study population

We obtained electronic health record data from patients registered with general practices contributing to the Clinical Practice Research Datalink (CPRD) during April 1, 2015, to March 31, 2016. The CPRD, a large database of anonymised electronic health records from UK primary care, contains patient-level data covering approximately 7% of the UK population [14]. CPRD data have been validated extensively and are representative of the UK population in terms of age, sex [14], and ethnic background [15]. We included patients of any age if their records were acceptable for research purposes (a data quality indicator provided by CPRD) and were registered at practices with continuous high-quality data reporting (CPRD defined up-to-standard) [16] at any time during the study period. We grouped patient data into their respective general practices.

The protocol was approved by the Independent Scientific Advisory Committee (ISAC) of the MHRA (ISAC protocol number 17_06R). Ethics approval for observational research using the CPRD with approval from ISAC was granted by a National Research Ethics Service committee (Trent MultiResearch Ethics Committee, REC reference number 05/MRE04/87).

### Included tests

We examined 44 specific tests (28 laboratory, 11 imaging and 5 other miscellaneous tests). The tests were chosen because they are commonly used tests or included in the guidelines or in the Quality Outcomes Framework (QOF) (Additional file 1: "Extended included tests" section).

We grouped tests into their respective general practices, via their practice identification number. To avoid double counting, if the same code was recorded multiple times for the same patient on the same day, it was counted as only one test. Similarly, codes likely referring to the same test, or separate components of a single test (e.g. individual components of a full blood count), were grouped and counted as one test.

### Statistical analysis

To identify which test was subject to the greatest between-practice variation in its use, we calculated, for each 44 tests, an unadjusted coefficient of variation (CoV) and then an adjusted CoV.

To calculate the unadjusted coefficient of variation, we did the following: we initially determined the number of tests ordered from each practice from April 1, 2015, to March 31, 2016. We then calculated the total person-years of observation for each general practice. Patients alive and registered for the entire year contributed 1 person-year of observation to the total. Patients who were born, died, registered, or deregistered during the year were included, but their contribution to the person-year calculation was adjusted proportionately (e.g. a patient who was registered and alive for only 6 months contributed 0.5 person-years).

We then calculated the mean unadjusted rate of use for each specific test across all 444 general practices; we also calculated the corresponding standard deviation. We used these two numbers to calculate the unadjusted CoV (standard deviation/mean × 100) [17]. The use of CoV facilitates a direct comparison of the variation in use between tests controlling for differences in sample size. It is expressed as a percentage (the ratio of the standard deviation to the mean), with larger percentages reflecting greater variation.

To calculate the adjusted CoV, we constructed a generalised linear model with Poisson errors to estimate the number of tests ordered from each general practice adjusted for practice differences in patient age, sex and deprivation. We constructed 44 Poisson models for each test. The age covariate represents the median age of each general practice, the sex covariate represents the proportion of female patients in each practice and the deprivation covariate represents the practice-level Index of Multiple Deprivation (IMD) deciles. We constructed Poisson models to adjust for differences in patient demographics (age, sex and IMD) between general practices. We did not construct Poisson models to compare the predictive ability of patient demographics on the rates of test use.

We then calculated the adjusted rate of use for each test in every general practice by dividing the adjusted number of tests by the person-years for each general practice (the same process we followed to calculate the unadjusted rate of use). The adjusted rates were used to calculate the adjusted CoV for each test, as described above [17]. We ranked tests according to their CoV. We present both the unadjusted and adjusted CoV in Additional file 1, but only the adjusted CoV in the main manuscript.

To identify the tests that had both a high rate of use and a high CoV, we calculated the overall median rate of test use and the overall median adjusted CoV. We then classified tests into four categories: (1) high variability, low rate, (2) high variability, high rate, (3) low variability, low rate or (4) low variability, high rate. These categories reflect a test’s measure in relation to the median value, e.g. high variability, low rate refers to tests with a coefficient of variation above the median coefficient of variation, but a rate of test use below the median rate of test use.

### Role of the funding source

This study was funded by an independent grant from the National Institute for Health Research (NIHR) School of Primary Care Research (Grant reference number 386) and the Primary Care Research Trust. Independent expert peer reviewers provided feedback on the grant application underpinning this study but had no further role in study design, data collection, analysis, interpretation or drafting of the manuscript.

## Results

Data from 444 general practices contributed to the CPRD from April 1, 2015, to March 31, 2016. In total, 16,496,218 tests were ordered for 4,078,091 patients over 3,311,050 person-years. The median age of patients was 40 (IQR 21 to 58) and the median percentage of females was 50.6% (IQR 49.7 to 51.3%). The total number of patients that each practice contributed varied (median 6955 (IQR 4374 to 9905), and deprivation differed between included general practice, ranging from 1 to 10 (median 6 (IQR 4 to 8)).

### Tests with the most variation in use

**Fig. 1:**
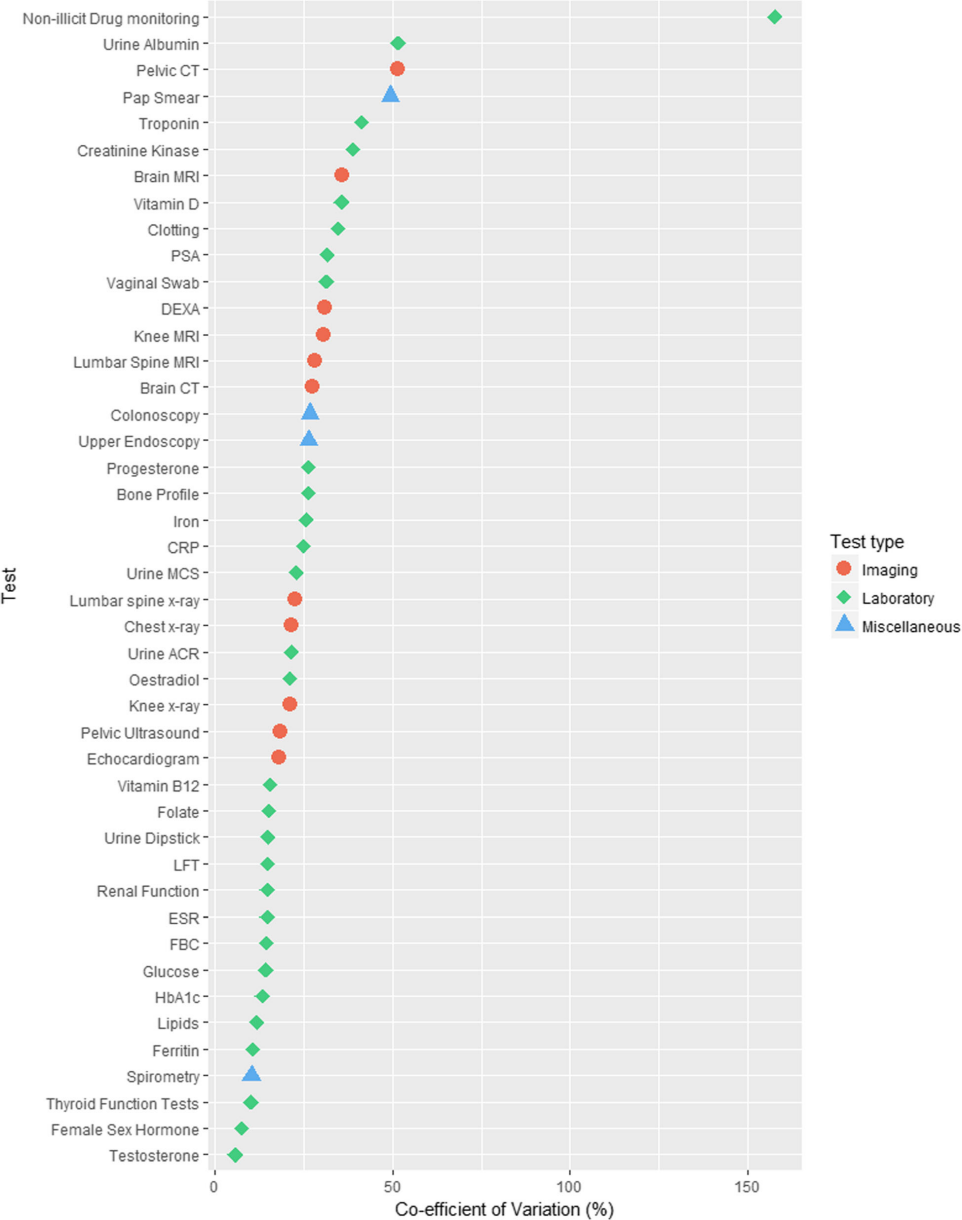
Rank order of variability of tests, adjusted for age, sex and deprivation. CT = Computer Tomography, MRI = Magnetic Resonance Imaging, PSA = Prostate Specific Antigen, DEXA = Dual-energy X-ray absorptiometry, CRP = C-reactive Protein, MCS = Microscopy, culture and sensitivities, ACR = Albumin-creatinine ratio, ESR = Erythrocyte sedimentation rate, LFT = Liver Function Tests, FBC = Full Blood Count

Figure 1 shows the rank order of the most to least variable tests. The adjusted CoV varied from 158% (95%CI 153 to 163%) for non-illicit drug monitoring tests (urine, blood or serum, for instance serum digoxin or lithium) to 5.6% (95%CI 5.4 to 5.8%) for testosterone tests. Urine microalbumin (52% (95%CI 49.9 to 53.2%)), pelvic CT (51% (95%CI 50 to 53%)) and Pap smear (49% (95%CI 48 to 51%) were the second, third and fourth most variable tests. Drug monitoring, pelvic CT and Pap smear tests were also the most variable laboratory, imaging and miscellaneous tests respectively. The median coefficient of variation was 22.7% (IQR 14.8 to 31.0%). These measures represent the between-practice variation in test ordering adjusted for age, sex and deprivation differences between practices (Additional file 1: Table S1).

**Fig. 2:**
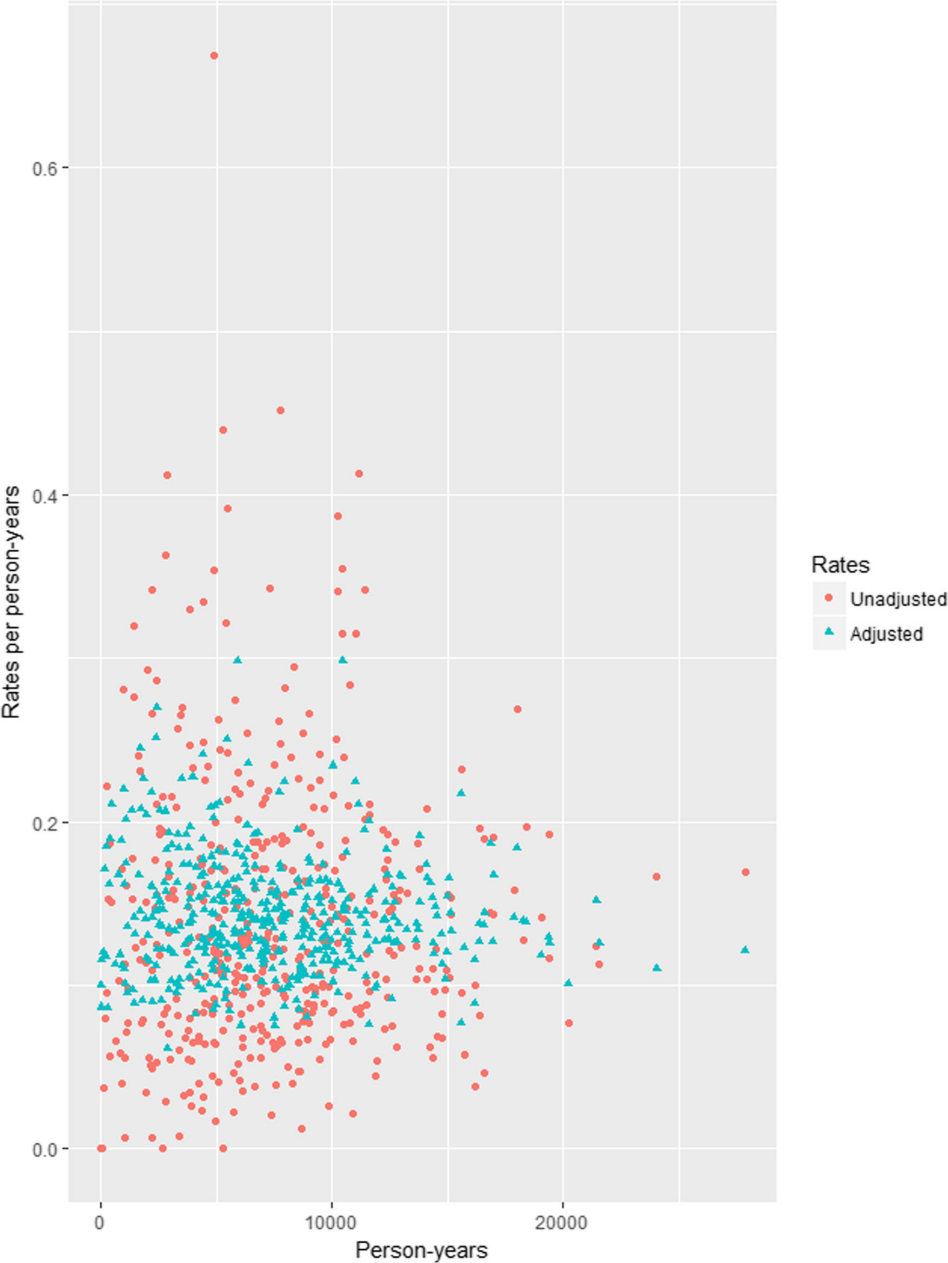
Adjusted and unadjusted rates for C-reactive Protein (CRP) use. All 444 practices are represented by one red and one blue data point

Additional file 1: Figure S1 shows the adjusted and unadjusted coefficients of variation for the 44 specific tests, and Additional file 1: Table S1 presents the difference between adjusted and unadjusted coefficients of variation for each specific test. Figure 2 shows an example of the adjusted and unadjusted rate of test use (CRP), measured against the respective person-years. This figure shows how the rates of CRP use for each 444 general practices is adjusted in accordance to their practice demographic (age, sex and deprivation). Similar graphs for the other 43 tests are displayed in Additional file 1 (page 10 onwards).

### Tests with a high rate of use and high variability of use

**Fig. 3:**
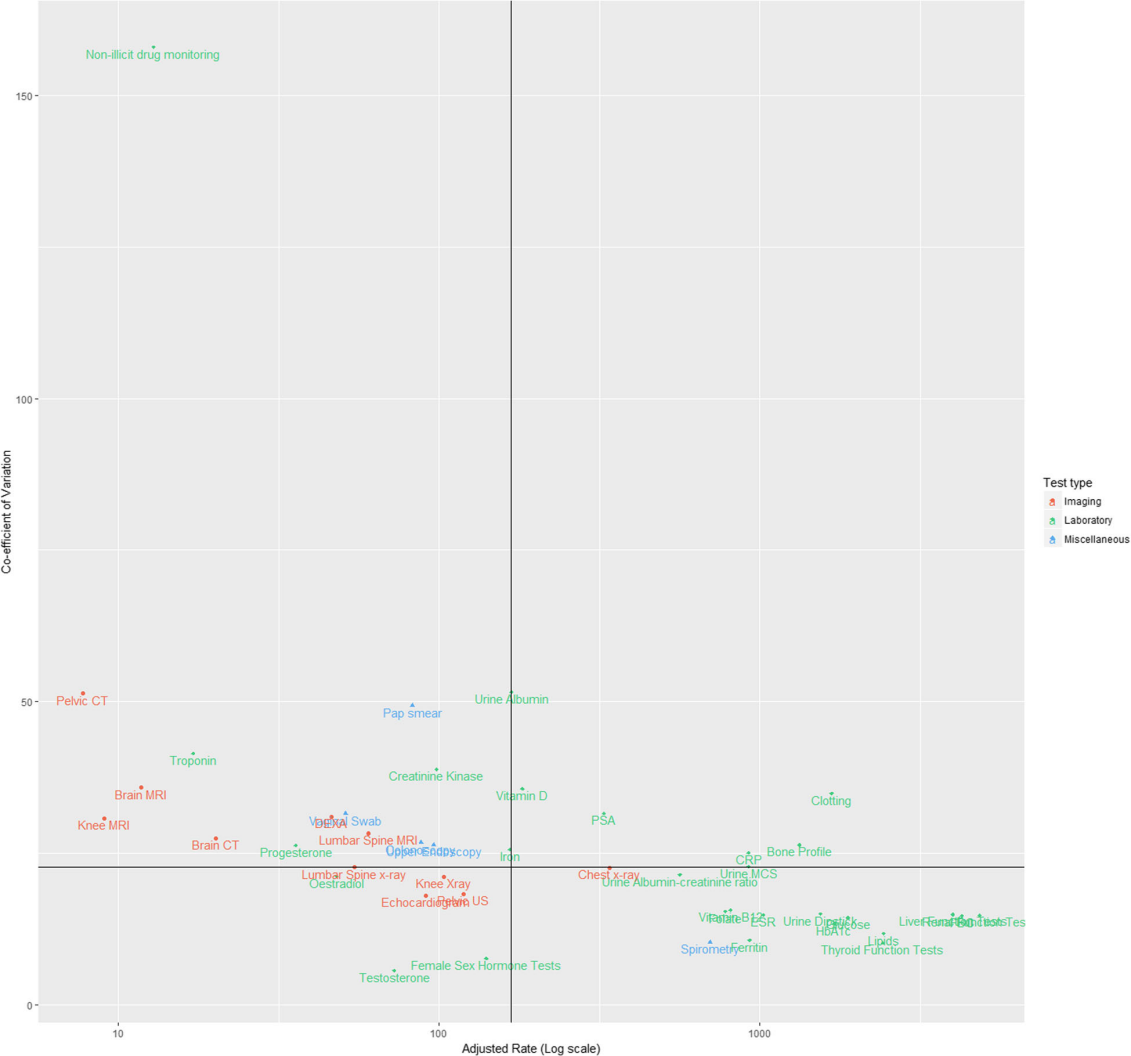
Variability and rates of tests. The vertical line represents the median rate of test use and the horizontal line represents the median coefficient of variation. Median rate (vertical line) = 167.3. Median CoV (horizontal line) = 22.7

Figure 3 plots the adjusted coefficient of variability of each test against its respective rate. The median rate was 167.3 (per 10,000 person-years) and the median CoV was 22.7. Most tests were classified as high variability, low rate (*n* = 15, 34%) or low variability, high rate (*n* = 15, 34%). Seven tests were classified as high variability and high rate (clotting, vitamin D, urine albumin, prostate-specific antigen (PSA), bone profile, urine MCS and C-reactive protein (CRP)). The remaining seven tests were classed as low variability and low rate.

Four miscellaneous tests were in the same category (high variability, low rate: colonoscopy, upper endoscopy, Pap smear and vaginal swab), with only one (spirometry) classed as low variability, high rate. Ten of the 11 imaging tests had rates of use below the median; four of these tests were classified as low variability and low rate (pelvic ultrasound, lumbar X-ray, knee X-ray and echocardiogram), while the other six were classified as high variability, low rate (pelvic CT, knee MRI, DEXA, brain MRI, brain CT and lumbar spine MRI). Only one imaging test had an adjusted rate above the median (chest X-ray), which was classified as low variability, high rate. Additional file 1: Table S2 shows the classification of all tests.

## Discussion

We present a ranking of 44 primary care tests based on the between-practice variation in their use. We analysed over 16 million tests from 444 general practices and ranked tests by their adjusted coefficient of variation. The test subject to the greatest variation was non-illicit drug monitoring tests (urine, blood or serum), urine microalbumin, pelvic CT, and Pap smear. We also identified seven tests with both a rate of ordering and a coefficient of variation above average: clotting, vitamin D, urine albumin, PSA, bone profile, urine MCS and CRP.

### Strengths and limitations in relation to previous research

Our analysis adjusted for demographic differences between practices; however, there may be valid reasons to explain the residual variation we present. Previous work has suggested that differences in disease prevalence, patient choice, data artefact (differences in data quality), resource availability, local policy and guidelines, and service configurations may also contribute to variation in healthcare resource use [10]. Other previous research suggests further reasons, not all justifiable. The influence of local key-opinion leaders [18]—such as a hospital consultant preferring a one test over another—and the variation in management of uncertainty among general practitioners [19] have both been suggested as contributors.

We used the conventional statistical analysis for count data, a Poisson regression model. However, we used the outputs of this model in a less conventional manner. The aim of this paper was to determine which tests were subject to the most between-variation practice in their use once patient demographics between practices had been accounted for. We did not use the Poisson models to determine and compare the predictive ability of our covariates (patient demographics). As is expected when analysing health care data [20], the model accounted for some, but not all, of the variation in test use. This residual variation—“overdispersion”—represents the variation in test use once patient demographics between practices had been accounted for. We ranked tests by their residual variation; variation in use that persisted despite adjustment of patient demographic differences between practices.

A strength of our study is our examination of all types of tests (imaging, laboratory and miscellaneous), inclusion of many tests and our use of the appropriate statistical methods to quantify variation. One previous study has presented a ranking of primary care tests on their between-practice variation [13]. This study only examined laboratory tests and included a smaller number of tests [21] in a smaller sample of patients. This study also ranked test variation by their standard deviation (SD). We preferred CoV to SD because SD is affected by the rate of testing (sample size). We found that tests that are most commonly ordered are more likely to have higher standard deviations. The use of SD to rank the between-practice variation may make tests that are ordered more commonly appear to have higher between-practice variation. A limitation of our use of CoV is that it may overestimate variation in low ordering tests; to try and mitigate this, we presented both the tests with the greatest between-practice variation and the tests with a rate of ordering and a CoV above average. We believe tests with both a high CoV and high rate of ordering should be the focus of future academic and policy work.

A further strength of our study is the use of high-quality, validated electronic health record data and the identification of tests that are subject to the greatest between-practice variation. Most previous research exploring geographical variation in healthcare resource use has focused on identifying regions that order a greater or lesser number of tests or treatments compared to the national average [11, 22–25].

### Implications for practice and policy

The wide between-practice variation in test use we present is unlikely to be explained entirely by clinical indication. We present a list of common and important primary care tests ranked by their between-practice variation. Policy makers must decide if the residual variation we present is warranted, and if it is not, understand why this variation exists and what can be done to mitigate it. Between-practice variation and, more broadly, geographical variation have long been used to highlight potential over or underuse of healthcare resources [6, 24, 26, 27]. Our ranking of tests can direct policy makers to the primary care tests most likely subject to overuse—the use of a test when it will not result in patient benefit—or underuse—the failure to use a test when it would result in patient benefit. However, it should be noted the variation we present does not directly consider individual patient data nor the clinical indications for test use. As such, our results can be considered a potential, not definitive, indicator of over and underuse.

In some cases, there are content-specific reasons to explain the between-practice variation in test use. For instance, the notable between-practice variation in the use of clotting and drug monitoring tests may reflect regional differences in drug use. In UK primary care, there has been an increase in the use of novel oral anticoagulants (NOAC) (also known as direct acting oral anticoagulants (DOACs) [28]; from 2009 to 2015, there was a 17-fold increase in NOAC use [28]. However, there is marked geographical variation in their use [21]. This variation may reflect the non-specific NICE guidance; it states that patients with atrial fibrillation can be anti-coagulated with “apixaban, dabigatran, rivaroxaban or a vitamin K antagonist” [29]. However, this guidance is now out-of-date compared to more recent evidence. A 2017 systematic review and network meta-analysis concluded that “the risk of all-cause mortality was lower with all DOACs” and “several DOACs are of net benefit compared with warfarin” [30]. With clear guidance, reflecting the underlying evidence, it is plausible that geographical variation in clotting tests would diminish.

Similarly, the variability of drug monitoring tests is likely to be related to regional differences in disease prevalence. Drug monitoring tests include tests for tacrolimus, cyclosporin, salicylate, lamotrigine, lithium and gentamicin (among others). All of these tests, individually, had low rates of use. Lastly, it should be noted that tests can be directly wasteful, but can also contribute to healthcare costs indirectly, for instance via incidental imaging findings [31].

### Future research

It would be advantageous for future studies to investigate variation using a different unit of an analysis. We chose to investigate variation at a practice level; however, future studies could investigate variation at a patient level or at a regional level. We chose to investigate at a practice level as previous literature suggests practice-level factors contribute substantially to healthcare variation [10, 18]. Differences in disease prevalence, cultural attitude to tests and their risks, local key-opinion leaders, resource availability, local policy and guidelines, and service configurations have all been suggested as practice-level contributors to variation.

A similar analysis aggregated at a regional, rather than practice, level may provide further insight into unwarranted variation. It is plausible that our analysis at a practice level may be too sensitive to variation in disease prevalence; this may in part explain non-illicit drug testing as an outlier. However, the aggregation of data at a regional level may obfuscate true, unwarranted variation. Furthermore, the CPRD only allows practices to be identified at a broad regional level (e.g. within Wales). Conversely, future research that analyses data at an individual patient level may provide more nuanced insight into variation, but risks being overly sensitive, making the distinction between warranted and unwarranted variation more difficult. Nevertheless, we would welcome any further studies that adopted any of the aforementioned units of analyses.

Furthermore, beyond adjustment for demographic differences, we could not directly determine the appropriateness of the between-practice variation we noted. Future research studies should aim to determine if the tests with the greatest between-practice variation are also subject to the greatest underuse and overuse. This research should ideally involve individual patient data (IPD) either in the form of notes review, or IPD data audit [32], commonly against guidelines [33]. Finally, some of our team are involved in delivering OpenPathology.net [24]; an open data tool (like OpenPrescribing.net) that provides easy access to various analytic approaches identifying test-ordering behaviour in primary care. This tool will continue our work exploring temporal trends on a live interface.

## Conclusions

There is wide variation among commonly used tests, which is unlikely to be explained by clinical indication, and since £3 billion annually are spent on tests, this represents considerable resource use variation and inefficient management for the NHS.

## Additional file

Additional file 1: Additional methods and results. (DOCX 725 kb)

## References

[1] Kleinert Sabine, Horton Richard (2017). From universal health coverage to right care for health. The Lancet.

[2] Fisher Elliott S., Bynum Julie P., Skinner Jonathan S. (2009). Slowing the Growth of Health Care Costs — Lessons from Regional Variation. New England Journal of Medicine.

[3] Alderwick H, Robertson R, Appleby J, Dunn P, Maguire D. Better value in the NHS the role of changes in clinical practice; 2015. Available from: https://www.kingsfund.org.uk/sites/default/files/field/field_publication_file/better-value-nhs-Kings-Fund-July%202015.pdf. Accessed 15 May 2018.

[4] The King’s Fund. The NHS budget and how it has changed [Internet]. London; 2017. Available from: https://www.kingsfund.org.uk/projects/nhs-in-a-nutshell/nhs-budget. Accessed 7 Apr 2018

[5] Martin Anne B., Hartman Micah, Washington Benjamin, Catlin Aaron (2017). National Health Spending: Faster Growth In 2015 As Coverage Expands And Utilization Increases. Health Affairs.

[6] Brownlee S, Chalkidou K, Doust J, Elshaug AG, Glasziou P, Heath I, et al. Evidence for overuse of medical services around the world. Lancet. 2017;6736(16):1–13 10.1016/S0140-6736(16)32585-5 PMC5708862 28077234

[7] Hobbs F D Richard, Bankhead Clare, Mukhtar Toqir, Stevens Sarah, Perera-Salazar Rafael, Holt Tim, Salisbury Chris (2016). Clinical workload in UK primary care: a retrospective analysis of 100 million consultations in England, 2007–14. The Lancet.

[8] Centers for Disease Control and Prevention, National Center for Health Statistics. National Ambulatory Medical Care Survey: 2012 Summary Tables. 2012;5. Available from: http://www.cdc.gov/nchs/data/ahcd/namcs_summary/2010_namcs_web_tables.pdfAccessed 17 Mar 2018.

[9] Wennberg JE (2010). Tracking medicine.

[10] Appleby J, Raleigh V, Frosini F, Bevan G, Gao H, Lyscom T. Variations in health care: the good, the bad and the inexplicable; 2011. Available from: https://www.kingsfund.org.uk/sites/default/files/field/field_publication_file/Variations-in-health-care-good-bad-inexplicable-report-The-Kings-Fund-April-2011.pdf. Accessed 20 Apr 2018.

[11] NHS Right Care. Diagnostics: the NHS atlas of variation in diagnostic services; 2012. Available from: https://fingertips.phe.org.uk/documents/DiagnosticAtlas_FINAL.pdf. Accessed 22 Mar 2018.

[12] Lord Carter of Coles. Report of the review of NHS pathology services in England. 2006 [cited 2018 Jan 5]. Available from: https://webarchive.nationalarchives.gov.uk/20130123210254/http://www.dh.gov.uk/en/Publicationsandstatistics/Publications/PublicationsPolicyAndGuidance/DH_4137606. Accessed 5 Jan 2018.

[13] Busby J, Schroeder K, Woltersdorf W, Sterne JAC, Ben-Shlomo Y, Hay A (2013). Temporal growth and geographic variation in the use of laboratory tests by NHS general practices: using routine data to identify research priorities. Br J Gen Pract.

[14] Herrett E, Gallagher AM, Bhaskaran K, Forbes H, Mathur R, van Staa T, et al. Data resource profile: clinical practice research datalink (CPRD). Int J Epidemiol. 2015;44(3):827–36. 10.1093/ije/dyv098 PMC4521131 26050254

[15] Mathur R, Bhaskaran K, Chaturvedi N, Leon DA, vanStaa T, Grundy E (2014). Completeness and usability of ethnicity data in UK-based primary care and hospital databases. J Public Health (Oxf).

[16] Williams Tim, van Staa Tjeerd, Puri Shivani, Eaton Susan (2012). Recent advances in the utility and use of the General Practice Research Database as an example of a UK Primary Care Data resource. Therapeutic Advances in Drug Safety.

[17] Armitage P, Berry G. Statistical methods in medical research; Wiley 1994.

[18] Wennberg JE (2011). Time to tackle unwarranted variations in practice. BMJ.

[19] Morgan M, Jenkins L, Ridsdale L (2007). Patient pressure for referral for headache: a qualitative study of GP’s referral behaviour. Br J Gen Pract.

[20] Spiegelhalter D J (2005). Handling over-dispersion of performance indicators. Quality and Safety in Health Care.

[21] Millett D (2016). NOAC prescribing varies 16-fold between CCG areas. GP online.

[22] NHS Rightcare. The NHS atlas of variation in healthcare. 2015. Available from: https://fingertips.phe.org.uk/documents/Atlas_2015%20Compendium.pdf. Accessed 20 Mar 2018.

[23] The Australian Commission on Safety and Quality in Health Care. EndFragmentAustralian atlas of healthcare variation. 2013. Available from: https://www.safetyandquality.gov.au/atlas/. Accessed 20 Apr 2018.

[24] O’Sullivan JW, Heneghan C, Perera R, Oke J, Aronson JK, Shine B (2018). Variation in diagnostic test requests and outcomes: a preliminary metric for OpenPathology.Net. Sci Rep.

[25] Wennberg John E. (1999). Understanding Geographic Variations in Health Care Delivery. New England Journal of Medicine.

[26] Glasziou Paul, Straus Sharon, Brownlee Shannon, Trevena Lyndal, Dans Leonila, Guyatt Gordon, Elshaug Adam G, Janett Robert, Saini Vikas (2017). Evidence for underuse of effective medical services around the world. The Lancet.

[27] Chalmers K, Pearson S, Elshaug AG (2017). Quantifying low-value care: a patient- versus service-centric lens. BMJ Qual Saf.

[28] Loo SY, Dell’Aniello S, Huiart L, Renoux C (2017). Trends in the prescription of novel oral anticoagulants in UK primary care. Br J Clin Pharmacol.

[29] NICE. NICE guideline: Atrial Fibrillation: management. 2014. Available from: https://www.nice.org.uk/guidance/cg180/resources/atrial-fibrillation-management-pdf-35109805981381. Accessed 20 Mar 2018.

[30] López-López JA, Sterne JAC, Thom HHZ, Higgins JPT, Hingorani AD, Okoli GN, et al. Oral anticoagulants for prevention of stroke in atrial fibrillation: systematic review, network meta-analysis, and cost effectiveness analysis. BMJ. 2018;361:k2295. 10.1136/bmj.k2295 PMC5965379 29793960

[31] O’Sullivan JW, Muntinga T, Grigg S, Ioannidis JPA. Prevalence and outcomes of incidental imaging findings: Umbrella review. BMJ. 2018;361:k2387. 10.1136/bmj.k2387. 10.1136/bmj.k2387 PMC6283350 29914908

[32] O’Sullivan Jack W, Albasri Ali, Nicholson Brian D, Perera Rafael, Aronson Jeffrey K, Roberts Nia, Heneghan Carl (2018). Overtesting and undertesting in primary care: a systematic review and meta-analysis. BMJ Open.

[33] O'Sullivan J.W., Albasri A., Koshiaris C., Aronson J.K., Heneghan C., Perera R. (2018). Diagnostic test guidelines based on high-quality evidence had greater rates of adherence: a meta-epidemiological study. Journal of Clinical Epidemiology.

