# Supplementary material for: Practice variation in the use of tests in UK primary care: a retrospective analysis of 16 million tests performed over 3.3 million patient years in 2015/16
Source: BMC Med. 2018 Dec 20;16:229. doi: 10.1186/s12916-018-1217-1 (PMC6300913; doi:10.1186/s12916-018-1217-1)

**Additional file 1: Practice variation in the use of tests in UK primary care: a retrospective analysis of 16 million tests performed over 3.3 million patient years of care in 2015/16.**

**Contents**

Extended included tests 2

Table S1 4

Table S2 7

Figure S1: Adjusted and unadjusted coefficient of variation for each 44 tests 9

Adjusted and unadjusted coefficient of variation for each 44 tests (individual graphs) 10

# Extended included tests

Below we list the 44 tests grouped into test type (28 laboratory, 11 imaging and five other, miscellaneous tests)

*Laboratory tests*

- Non-Illicit Drug monitoring
- Urine Albumin
- Troponin
- Clotting
- Vitamin D
- Creatinine Kinase
- Prostate specific Antigen (PSA)
- Iron
- Progesterone
- Bone Profile
- Creatine Reactive Protein (CRP)
- Urine Microscopy culture sensitivities (MCS)
- Oestradiol
- Urine Albumin: Creatinine Ratio (ACR)
- HbA1c
- Vitamin B12
- Erythrocyte Sedimentation Rate (ESR)
- Folate
- Liver Function Tests (LFT)
- Full Blood Count (FBC)

*Imaging tests*

- Pelvic computer tomography (CT)
- Brain Magnetic resonance imaging (MRI)
- Knee MRI
- Dual-energy X-ray absorptiometry (DEXA)
- Lumbar Spine MRI
- Brain CT
- Lumbar spine x-ray
- Chest x-ray
- Knee x-ray
- Pelvic Ultrasound
- Echocardiogram

*Other, miscellaneous tests*

- Pap Smear
- Vaginal Swab
- Upper Endoscopy
- Colonoscopy

Twenty-five tests were selected as they met one of the following criteria: Specific guidance on their use in primary care is stated in one or more of the following guidelines/frameworks: Quality Outcomes Framework (QOF), National Institute of Health and Clinical Excellence (NICE), Choosing Wisely or NICE Do Not Do guidelines. They were one of the two most frequent laboratory or imaging tests ordered from Oxfordshire primary care (data attained directly from Oxford University Hospital (OUH)). This list was reviewed and confirmed by the patient and public involvement (PPI) representatives and suggestions from this group were also considered.

A further 19 tests were identified as part of data cleaning and in consultation with GPs as tests that are typically ordered as one test but return many results. For instance, a liver function test returns results for Alanine aminotransferase (ALT), Aspartarte aminotransferase (AST), Alkaline phosphatase (ALP) and Gamma glutamyl transferase (GGT) amongst others, all of which have individual Read codes and are recorded separately.

# Table S1

Table S1 Difference between adjusted and unadjusted coefficient of variation

| **Test** | **Coefficient of variation (adjusted)** | **Coefficient of variation (unadjusted)** | **Difference between unadjusted and adjusted co-efficients of variation** |
| --- | --- | --- | --- |
| Non-illicit Drug monitoring | 157.9% (152.8% to 163.1%) | 817.9% (813.0% to 822.9%) | 660.0% |
| Urine Albumin | 51.6% (49.9% to 53.2%) | 177.6% (176.1% to 179.2%) | 126.0% |
| Pelvic CT | 51.3% (49.6% to 53.0%) | 204.7% (203.1% to 206.3%) | 153.4% |
| Pap Smear | 49.3% (47.7% to 50.9%) | 225.3% (223.7% to 226.9%) | 176.1% |
| Troponin | 41.4% (40.1% to 42.8%) | 202% (200.6% to 203.3%) | 160.5% |
| Creatinine Kinase | 38.8% (37.5% to 40.1%) | 216.6% (215.4% to 217.8%) | 177.8% |
| Brain MRI | 35.8% (34.6% to 36.9%) | 143.1% (142.0% to 144.2%) | 107.3% |
| Vitamin D | 35.6% (34.4% to 36.7%) | 132.9% (131.6% to 134.1%) | 97.3% |
| Clotting | 34.8% (33.7% to 36.0%) | 79.5% (78.2% to 80.7%) | 44.6% |
| PSA | 31.5% (30.5% to 32.6%) | 59.5% (58.5% to 60.5%) | 28.0% |
| Vaginal Swab | 31.4% (30.4% to 32.5%) | 163.4% (162.3% to 164.4%) | 131.9% |
| DEXA | 30.9% (29.9% to 31.9%) | 98.5% (97.5% to 99.4%) | 67.5% |
| Knee MRI | 30.7% (29.7% to 31.7%) | 165.7% (164.7% to 166.8%) | 135.1% |
| Lumbar Spine MRI | 28.2% (27.3% to 29.2%) | 118.6% (117.7% to 119.5%) | 90.3% |
| Brain CT | 27.4% (26.5% to 28.3%) | 140.7% (140.0% to 141.5%) | 113.3% |
| Colonoscopy | 26.7% (25.8% to 27.6%) | 79.6% (78.9% to 80.4%) | 52.9% |
| Bone Profile | 26.3% (25.5% to 27.2%) | 73.8% (73.1% to 74.7%) | 45.4% |
| Progesterone | 26.3% (25.4% to 27.1%) | 139.2% (138.3% to 140.1%) | 112.9% |
| Upper Endoscopy | 26.4% (25.5% to 27.2%) | 84.7% (83.9% to 85.5%) | 58.3% |
| Iron | 25.6% (24.7% to 26.4%) | 204.9% (204.1% to 205.9%) | 179.4% |
| CRP | 25.0% (24.2% to 25.9%) | 59.5% (58.7% to 60.3%) | 34.5% |
| Urine MCS | 22.8% (22.0% to 23.5%) | 78.4% (77.7% to 79.1%) | 55.6% |
| Lumbar spine x-ray | 22.6% (21.9% to 23.4%) | 105.5% (104.7% to 106.3%) | 82.8% |
| Chest x-ray | 21.6% (20.9% to 22.3%) | 71.7% (71.0% to 72.5%) | 50.1% |
| Urine ACR | 21.4% (20.7% to 22.1%) | 53.3% (52.6% to 53.9%) | 31.8% |
| Knee x-ray | 21.1% (20.4% to 21.8%) | 71.4% (70.7% to 72.1%) | 50.3% |
| Oestradiol | 21.1% (20.4% t o 21.8%) | 122.5% (121.8% to 123.2%) | 101.4% |
| Pelvic Ultrasound | 18.3% (17.7% to 18.8%) | 111.1% (110.5% to 111.7%) | 92.9% |
| Echocardiogram | 17.9% (17.4% to 18.5%) | 86.3% (85.8% to 86.8%) | 68.4% |
| Vitamin B12 | 15.5% (15.0% to 16.1%) | 71.7% (71.2% to 72.1%) | 56.1% |
| Folate | 15.3% (14.8% to 15.8%) | 73.5% (73.0% to 74.0%) | 58.2% |
| Urine Dipstick | 14.9% (14.4% to 15.4%) | 54.7% (54.2% to 55.1%) | 39.7% |
| LFT | 14.8% (14.3% to 15.3%) | 33% (32.6% to 33.5%) | 18.2% |
| ESR | 14.7% (14.2% to 15.2%) | 77.9% (77.5% to 78.4%) | 63.2% |
| Renal Function | 14.7% (14.2% to 15.2%) | 31.2% (30.7% t o 31.6%) | 16.4% |
| FBC | 14.6% (14.1% to 15.1%) | 32.6% (32.2% to 33.1%) | 18.1% |
| Glucose | 14.3% (13.8% to 14.7%) | 56.9% (56.4% to 57.3%) | 42.6% |
| HbA1c | 13.3% (12.8% to 13.7%) | 52.4% (51.9% to 52.9%) | 39.2% |
| Lipids | 11.7% (11.3% to 12.1%) | 37.9% (37.5% to 38.3%) | 26.2% |
| Ferritin | 10.6% (10.2% to 11.1%) | 63.6% (63.2% to 63.9%) | 52.9% |
| Spirometry | 10.2% (9.9% to 10.5%) | 41.3% (40.9% to 41.6%) | 31.1% |
| Thyroid Function Tests | 10.1% (9.8% to 10.5%) | 33.7% (33.4% to 33.9%) | 23.5% |
| Female Sex Hormone | 7.5% (7.3% to 7.8%) | 42.3% (42.1% to 42.6%) | 34.8% |
| Testosterone | 5.6% (5.4% to 5.8%) | 45.8% (45.6% to 46.0% | 40.2% |

# Table S2

| **High Variability**  **Low Rate** | | | **High Variability**  **High Rate** | | |
| --- | --- | --- | --- | --- | --- |
| *Test* | *Co-efficient of Variation* | *Adjusted rate (per 10,000 person-years)* | *Test* | *Co-efficient of Variation* | *Adjusted rate (per 10,000 person-years)* |
| Iron | 25.6 | 166.3 | Clotting | 34.8 | 1676.7 |
| Creatinine Kinase | 38.8 | 98.2 | Bone Profile | 26.3 | 1331.5 |
| Upper Endoscopy | 26.4 | 96.3 | C-reactive Protein | 25.0 | 924.4 |
| Colonoscopy | 26.7 | 87.9 | Prostate Specific Antigen | 31.5 | 326.5 |
| Pap smear | 49.3 | 82.6 | Vitamin D | 35.6 | 182.0 |
| Lumbar Spine MRI | 28.2 | 60.3 | Urine Albumin | 51.6 | 168.4 |
| Pelvic CT | 51.3 | 7.8 | Urine MCS | 22.8 | 924.4 |
| Vaginal Swab | 31.4 | 51.0 |  |  |  |
| DEXA | 30.9 | 46.1 |  |  |  |
| Progesterone | 26.3 | 35.7 |  |  |  |
| Brain CT | 27.4 | 20.1 |  |  |  |
| Troponin | 41.4 | 17.1 |  |  |  |
| Non-illicit drug monitoring | 157.9 | 12.9 |  |  |  |
| Brain MRI | 35.8 | 11.8 |  |  |  |
| Knee MRI | 30.7 | 9.1 |  |  |  |
| **Low Variability**  **Low Rate** | | | **Low Variability**  **High Rate** | | |
| *Test* | *Co-efficient of Variation* | *Adjusted rate (per 10,000 person-years)* | *Test* | *Co-efficient of Variation* | *Adjusted rate (per 10,000 person-years)* |
| Female Sex Hormone Tests | 7.5 | 140.5 | Renal Function Tests | 14.7 | 4854.6 |
| Pelvic Ultrasound | 18.3 | 119.3 | FBC | 14.7 | 4272.1 |
| Knee X-ray | 21.1 | 103.6 | Liver Function Tests | 13.8 | 4002.8 |
| Echocardiogram | 17.9 | 91.0 | Lipids | 11.7 | 2434.2 |
| Testosterone | 5.6 | 72.5 | Thyroid Function Tests | 10.1 | 2424.0 |
| Oestradiol | 21.1 | 48.0 | Glucose | 14.3 | 1887.4 |
| Lumbar Spine x-ray | 22.6 | 54.3 | HbA1c | 13.3 | 1711.2 |
|  |  |  | Urine Dipstick | 14.9 | 1548.2 |
|  |  |  | ESR | 14.7 | 1027.1 |
|  |  |  | Ferritin | 10.6 | 930.5 |
|  |  |  | Vitamin B12 | 15.5 | 811.7 |
|  |  |  | Urine Albumin-creatinine ratio | 21.4 | 564.4 |
|  |  |  | Folate | 15.3 | 780.7 |
|  |  |  | Spirometry | 10.2 | 700.7 |
|  |  |  | Chest x-ray | 22.6 | 339.3 |

# Figure S1: Adjusted and unadjusted coefficient of variation for each 44 tests


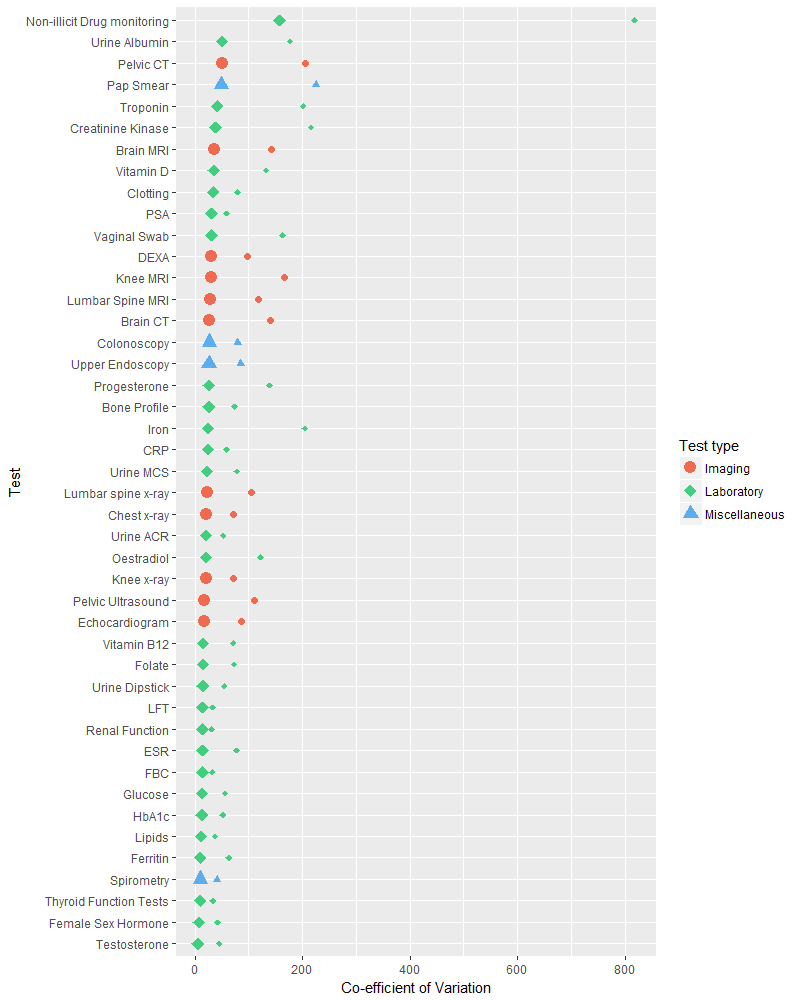


Figure S1 Adjusted (larger symbol) and unadjusted (smaller symbol) coefficient of variation:
Larger symbols show adjusted coefficients. CT = Computer Tomography, MRI = Magnetic Resonance Imaging, PSA = Prostate Specific Antigen, DEXA = Dual-energy X-ray absorptiometry, CRP = C-reactive Protein, MCS = Microscopy, culture and sensitivities, ACR = Albumin-creatinine ratio, ESR = Erythrocyte sedimentation rate, LFT = Liver Function Tests, FBC = Full Blood Count.

# Adjusted and unadjusted coefficient of variation for each 44 tests (individual graphs)


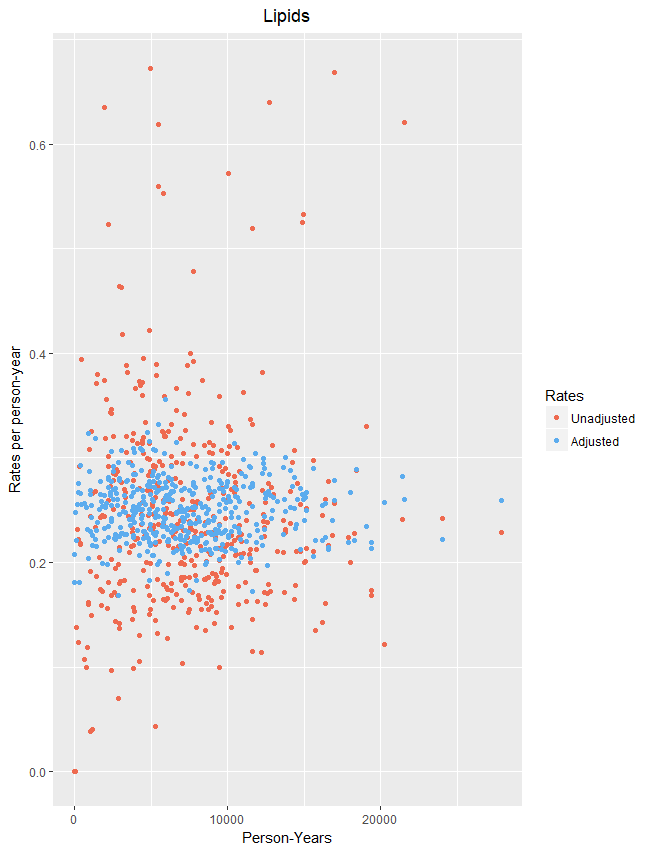

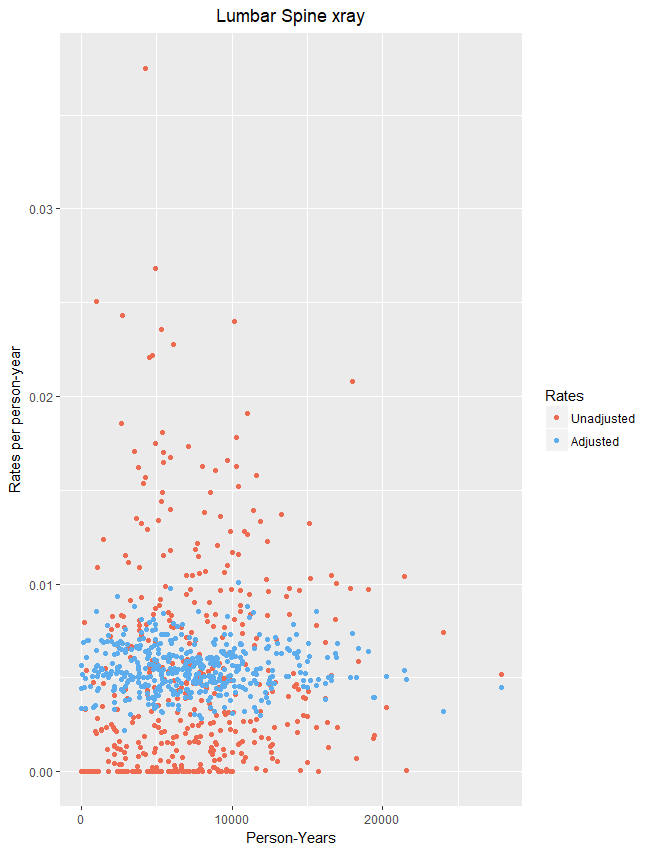

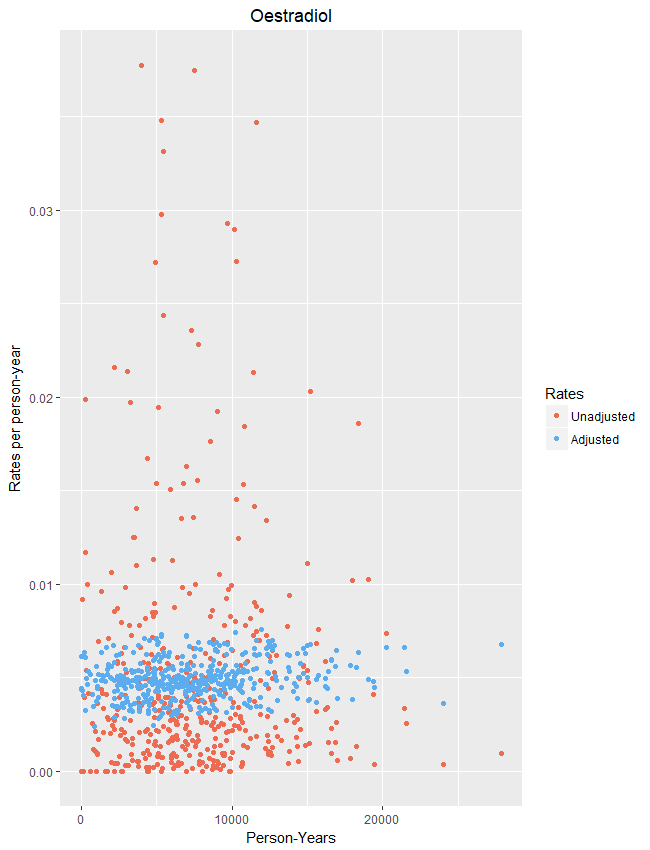

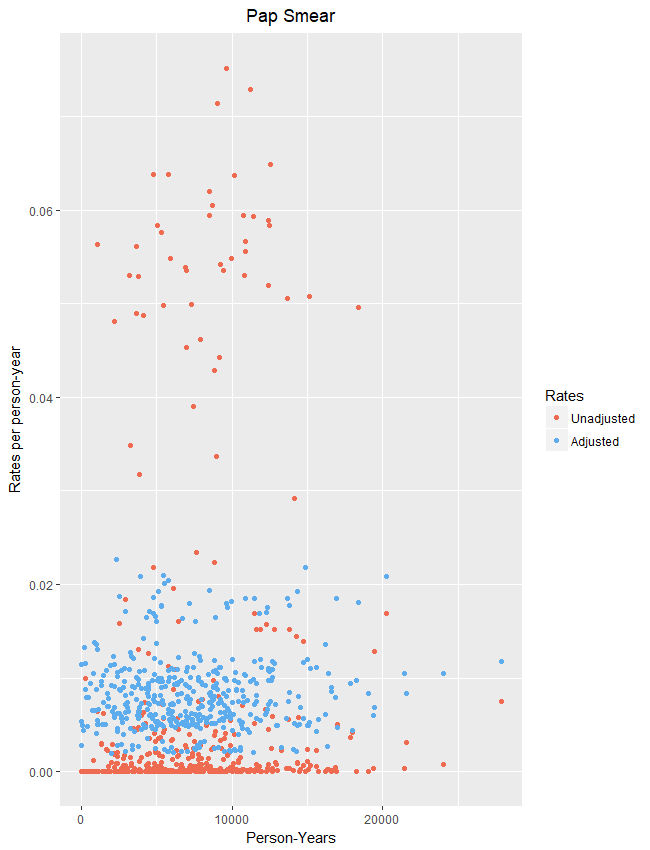

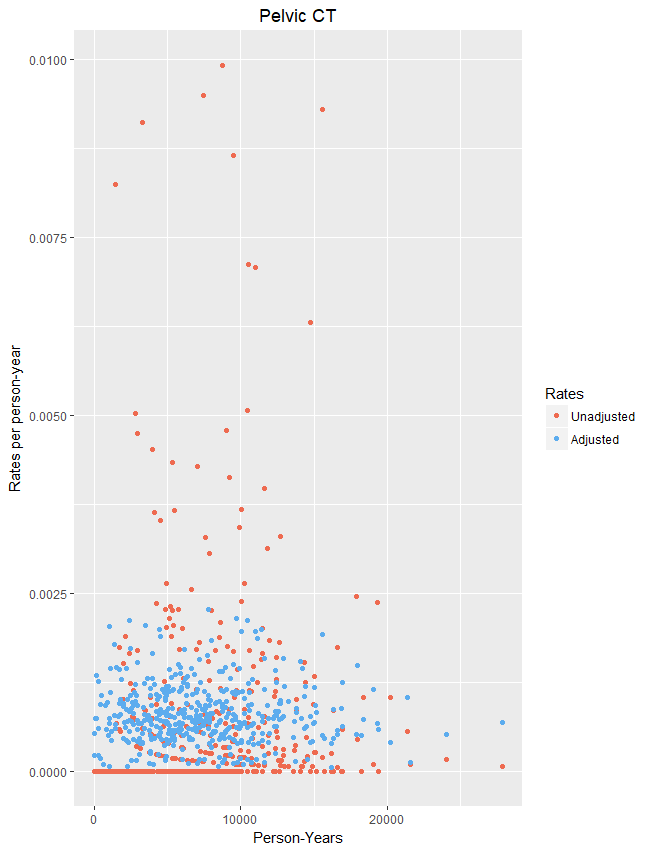

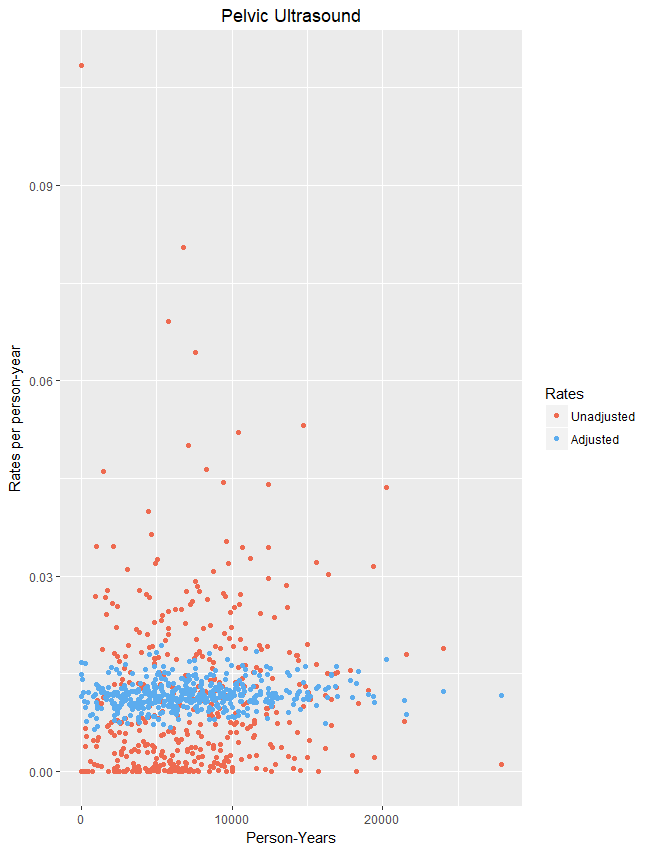

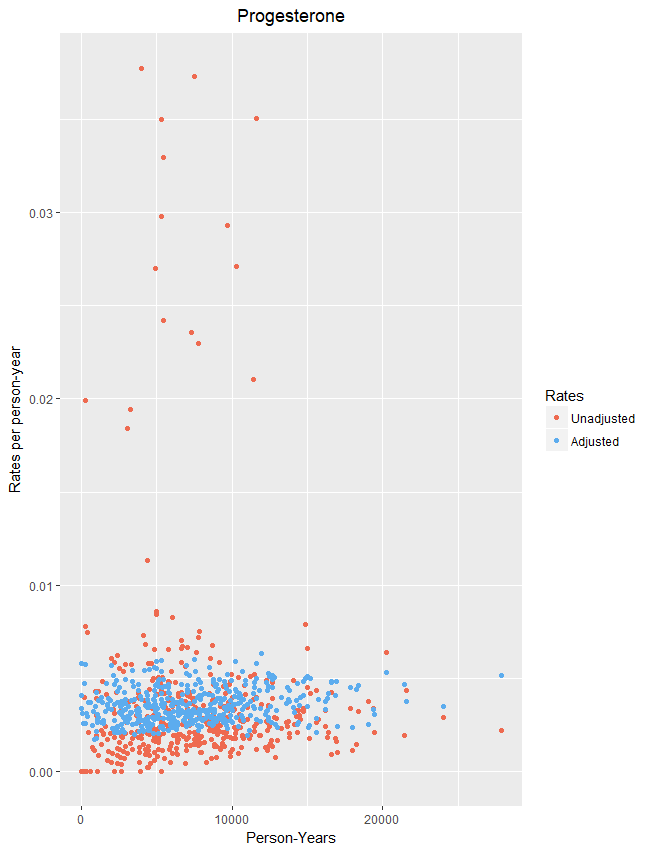

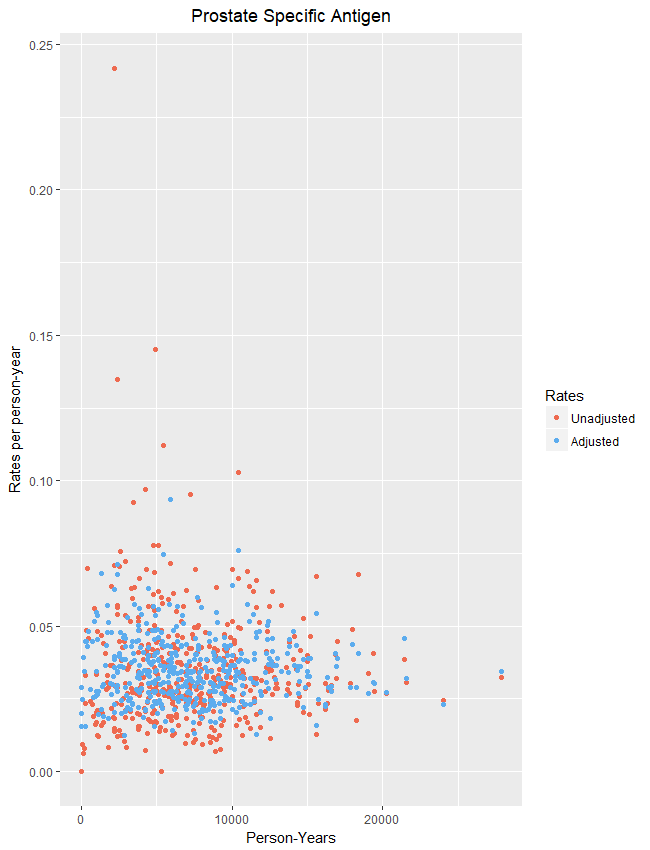

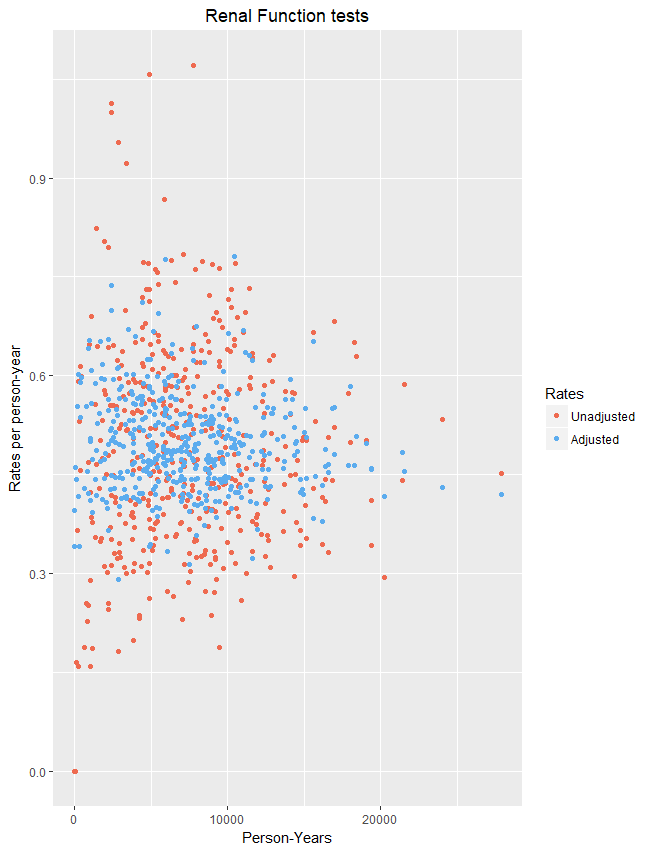

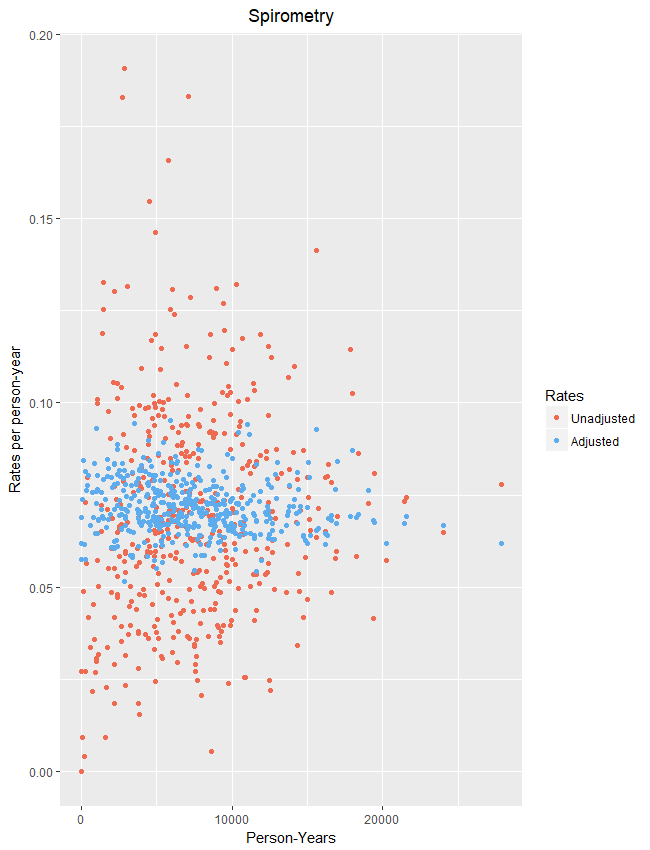

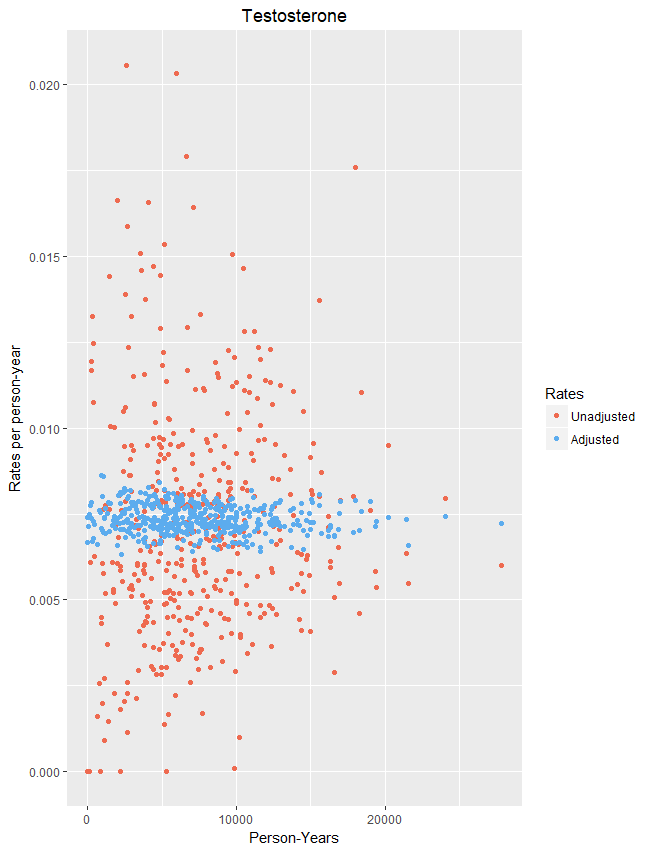

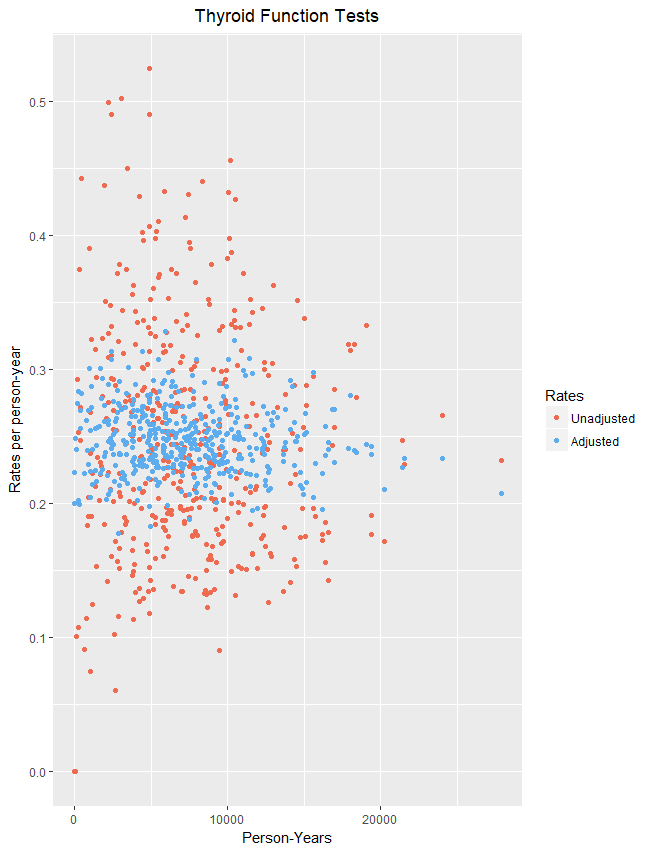

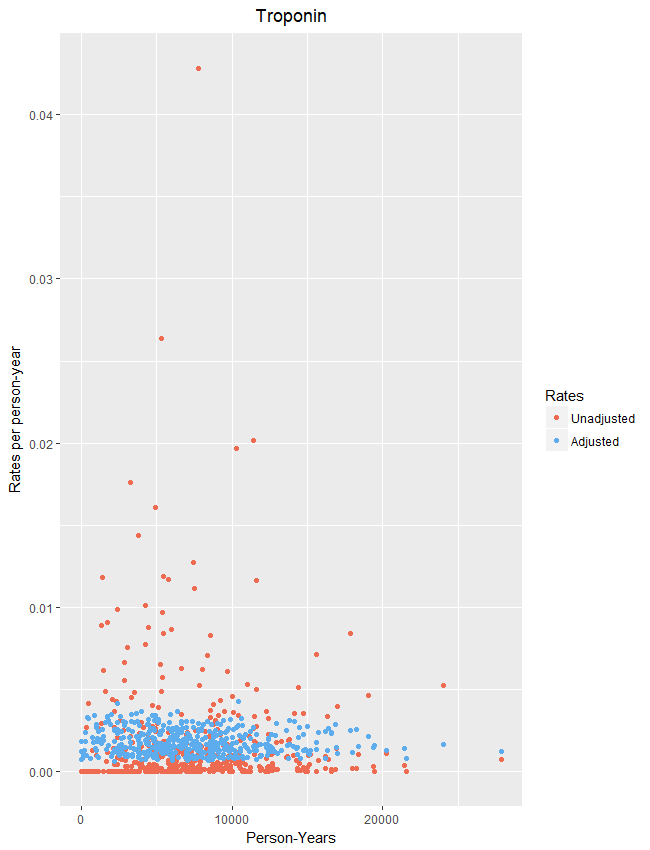

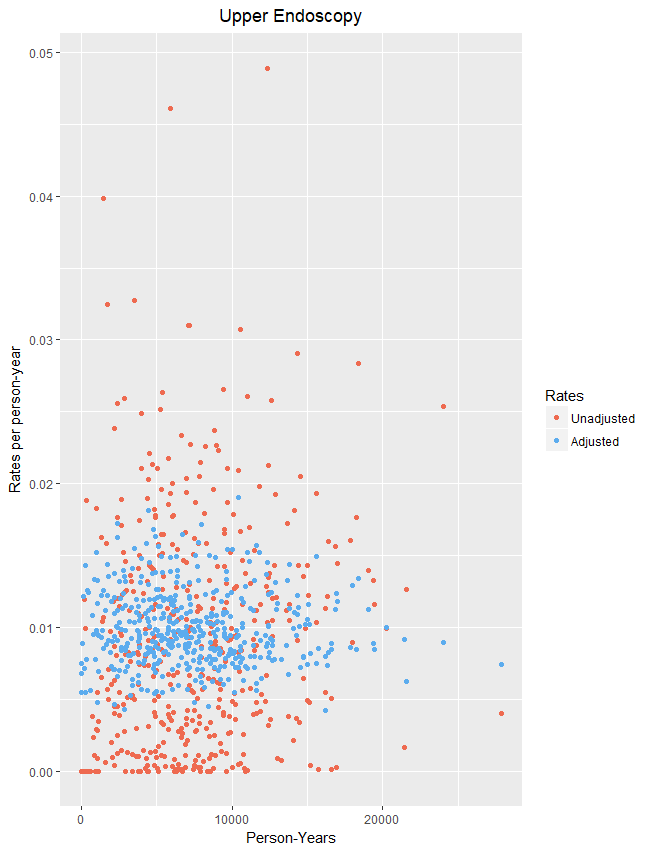

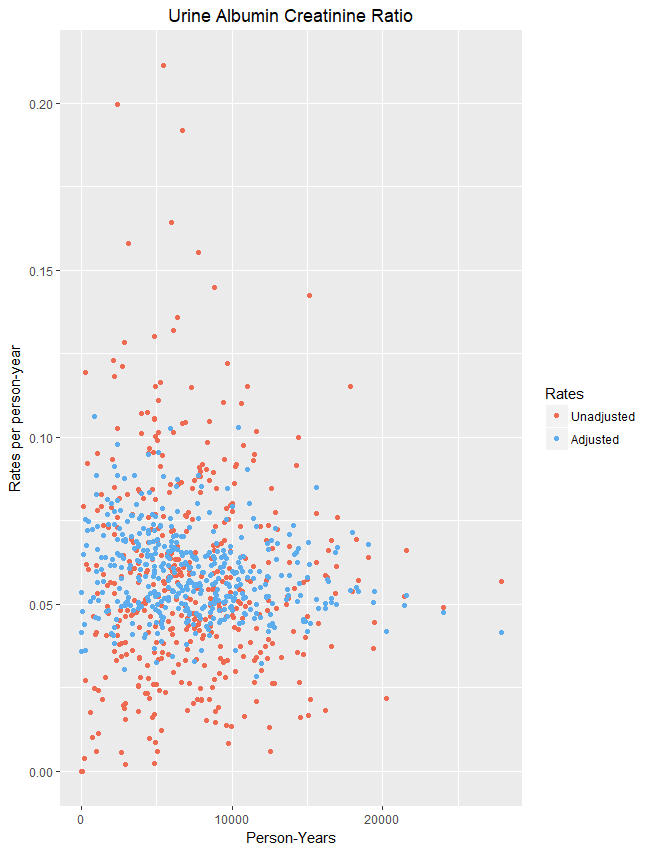

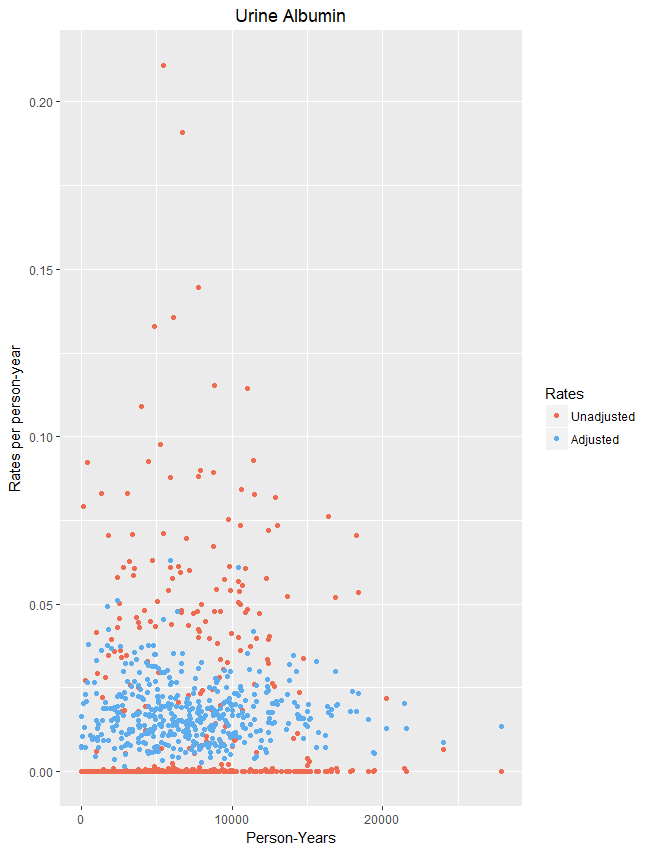

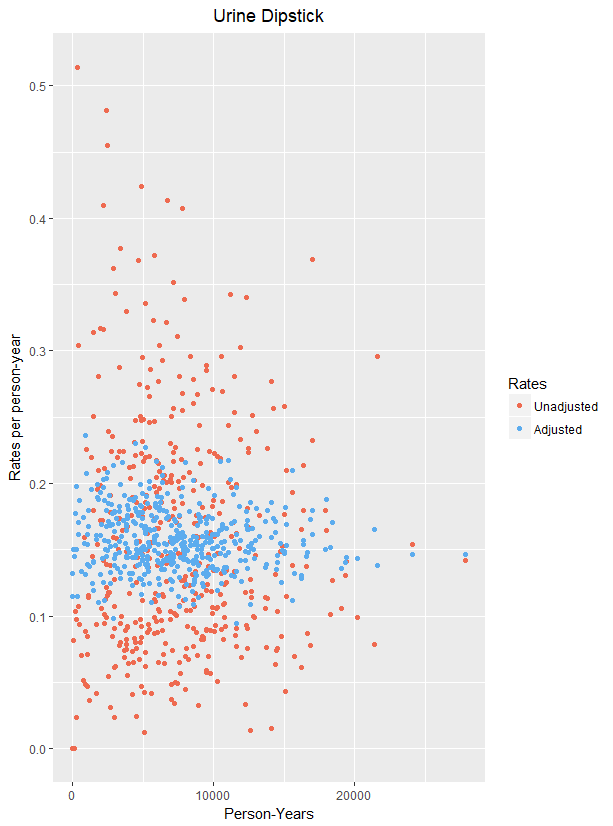

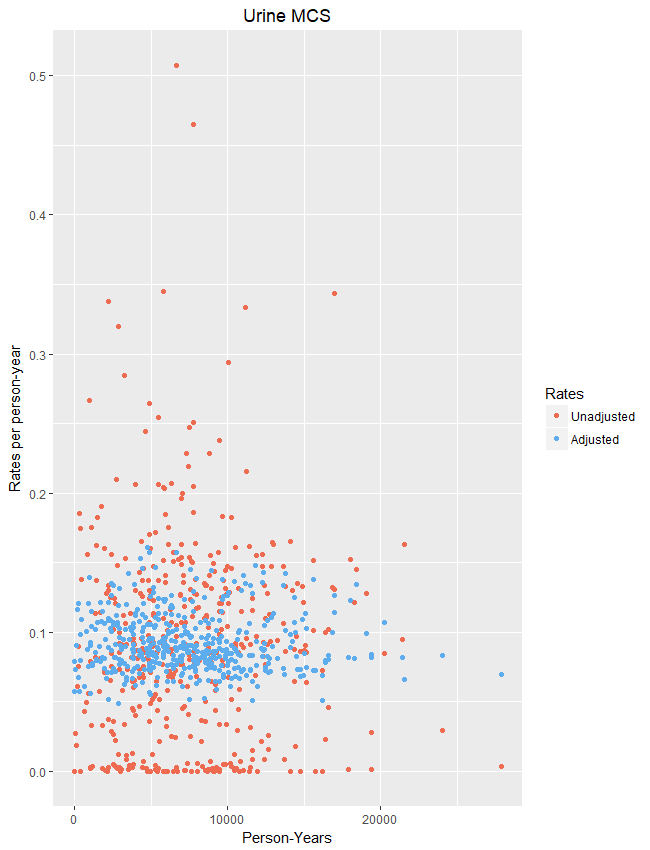

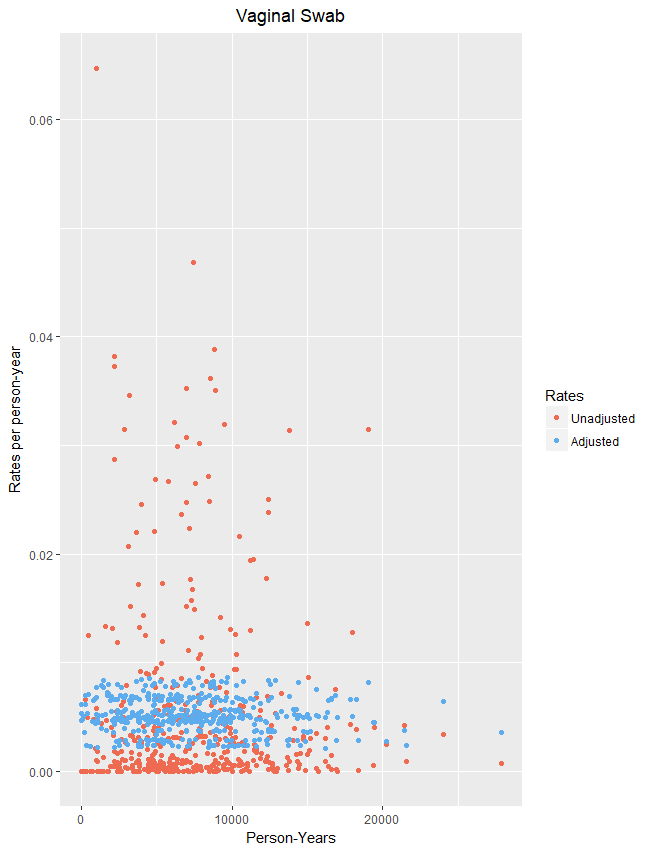

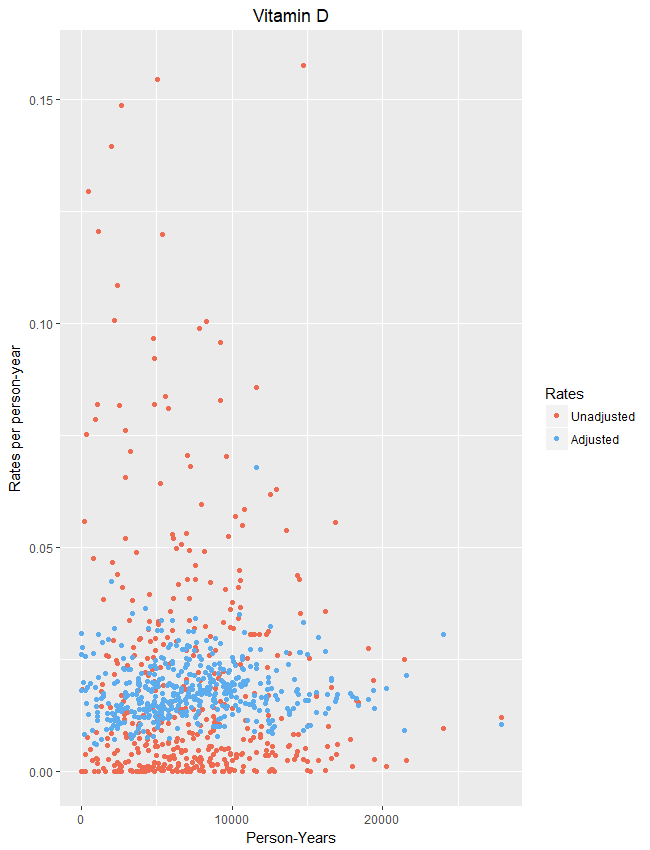

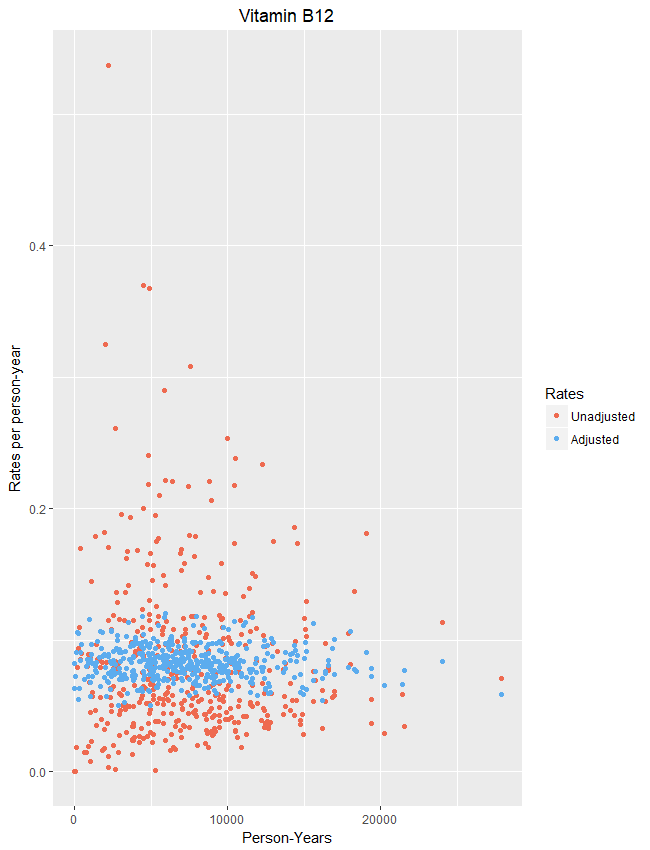

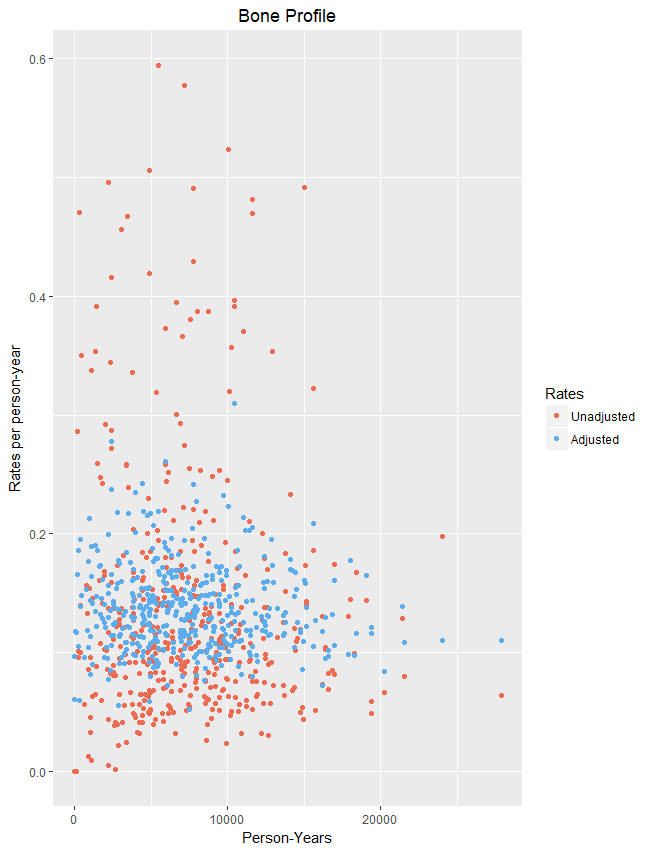

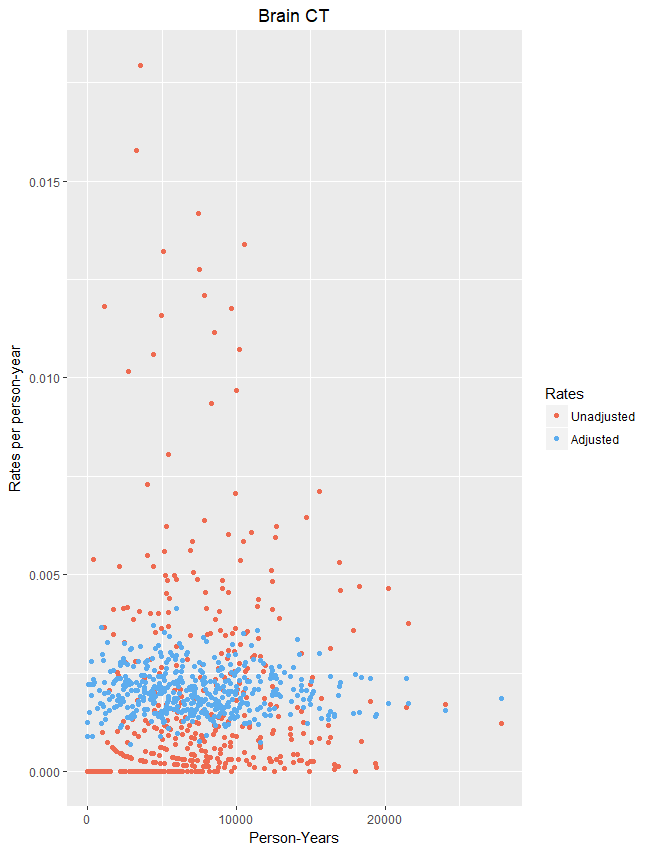

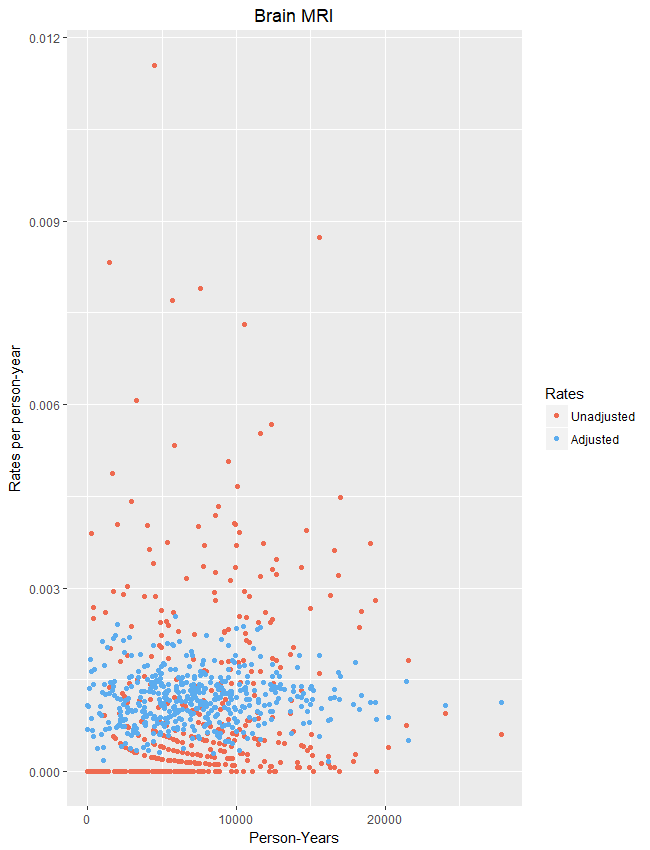

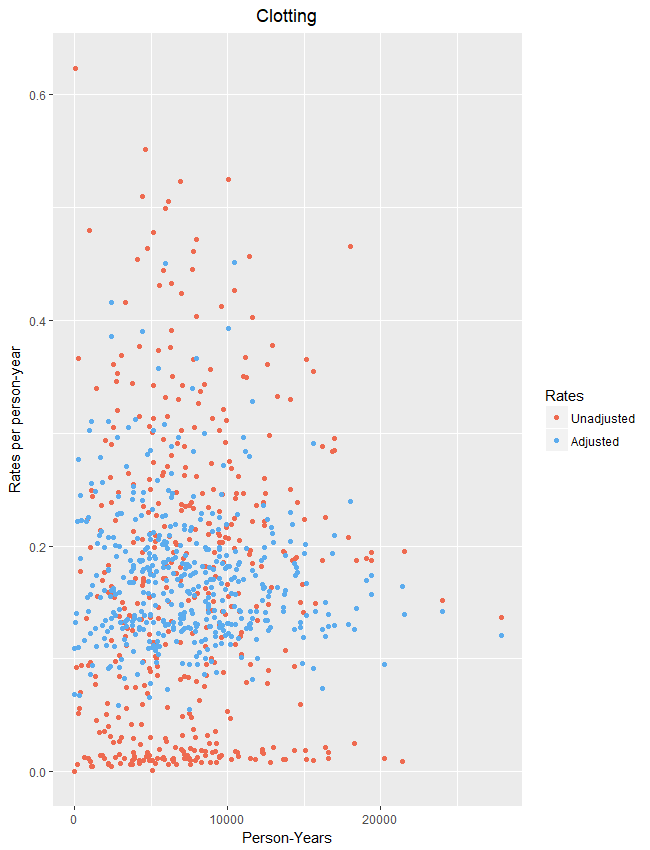

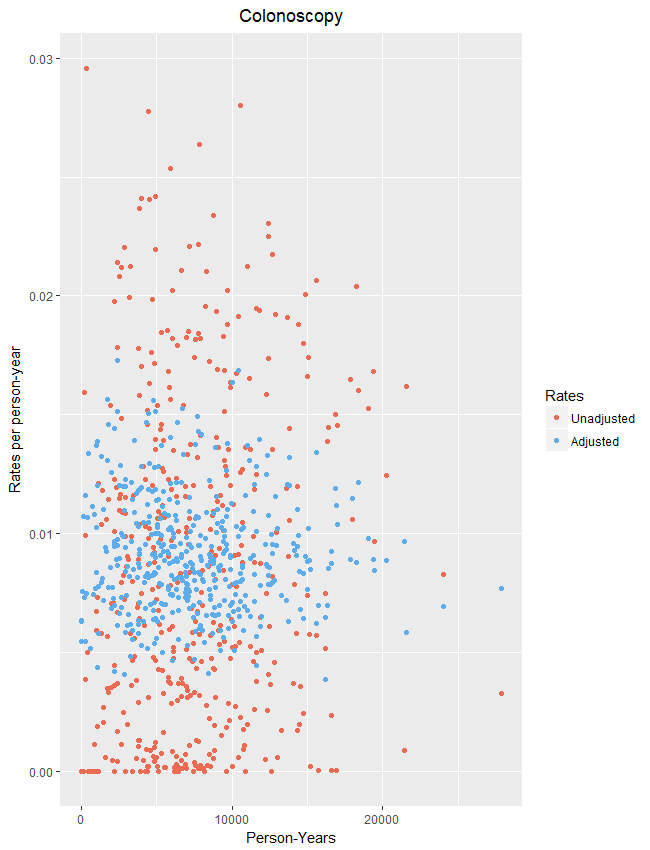

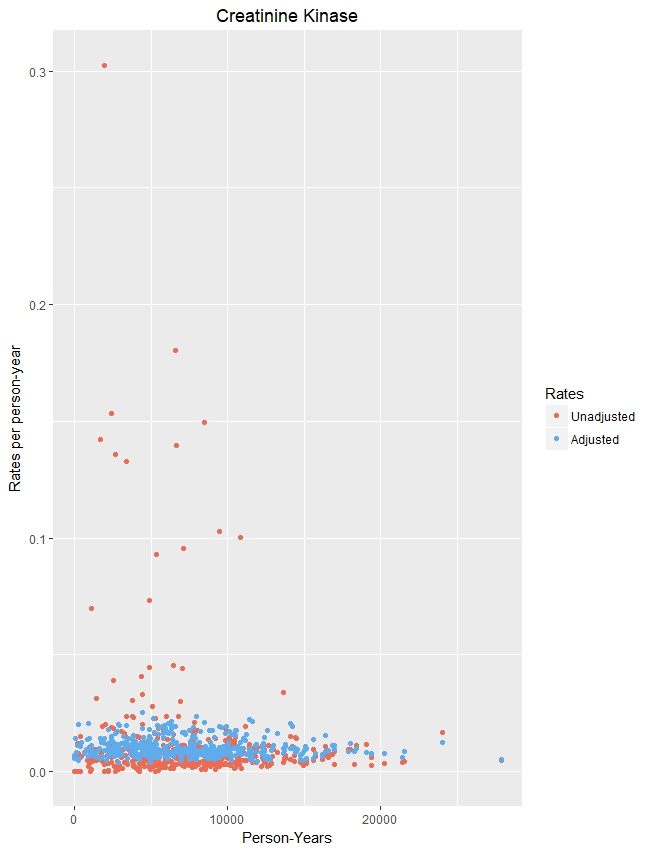

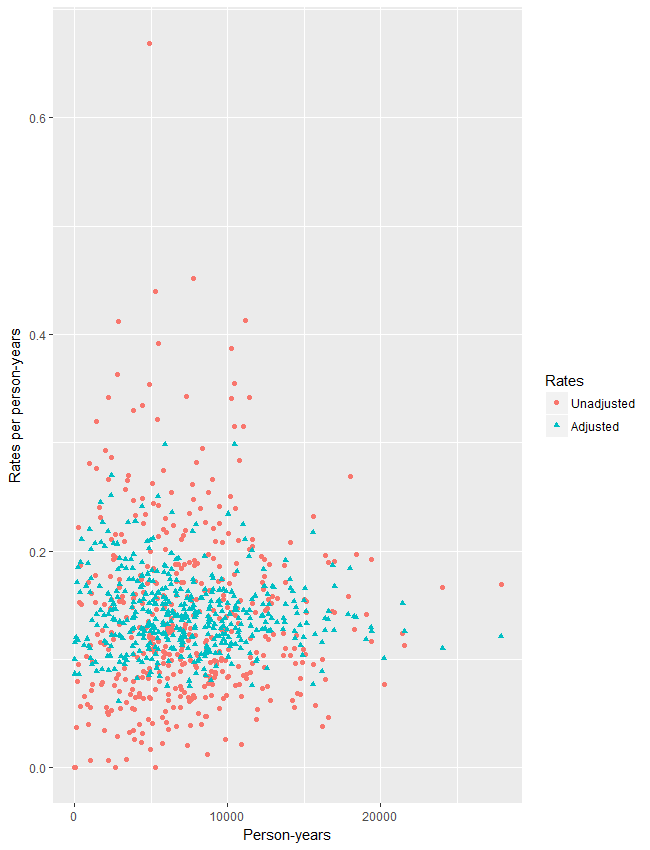

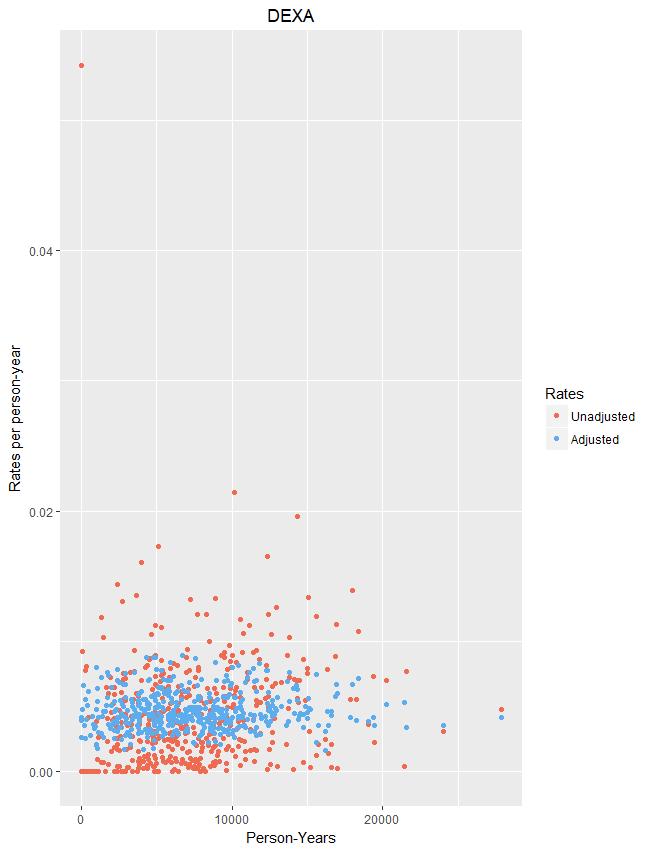

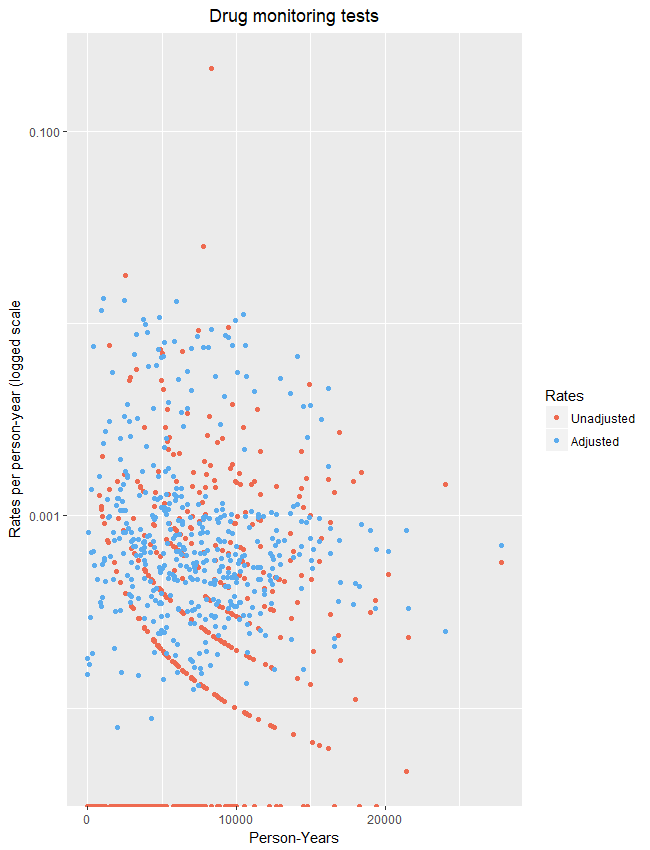

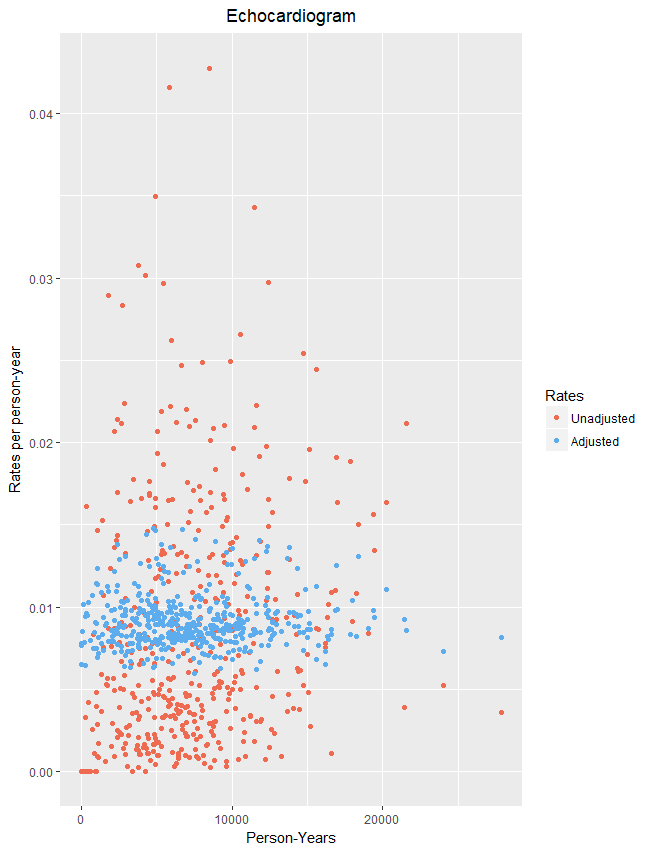

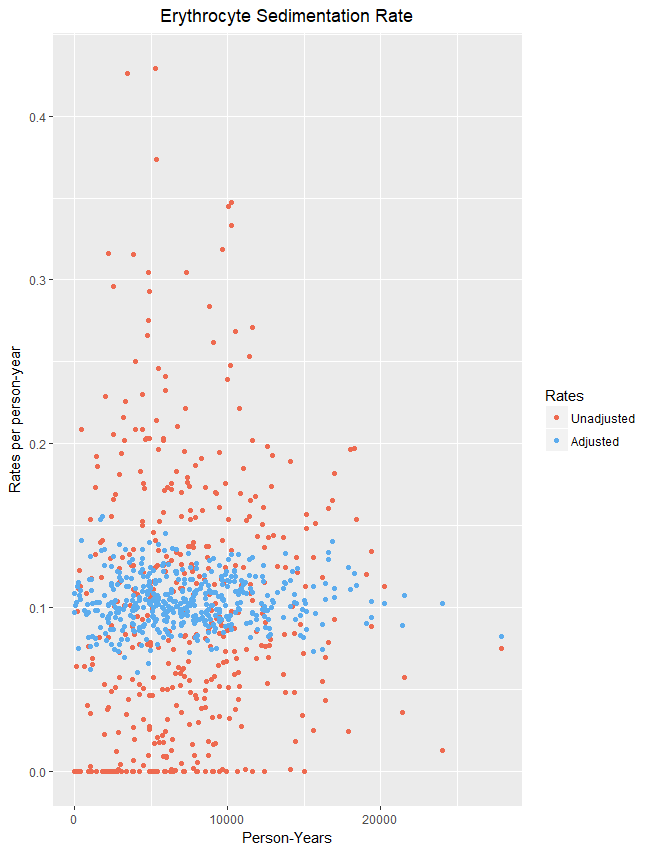

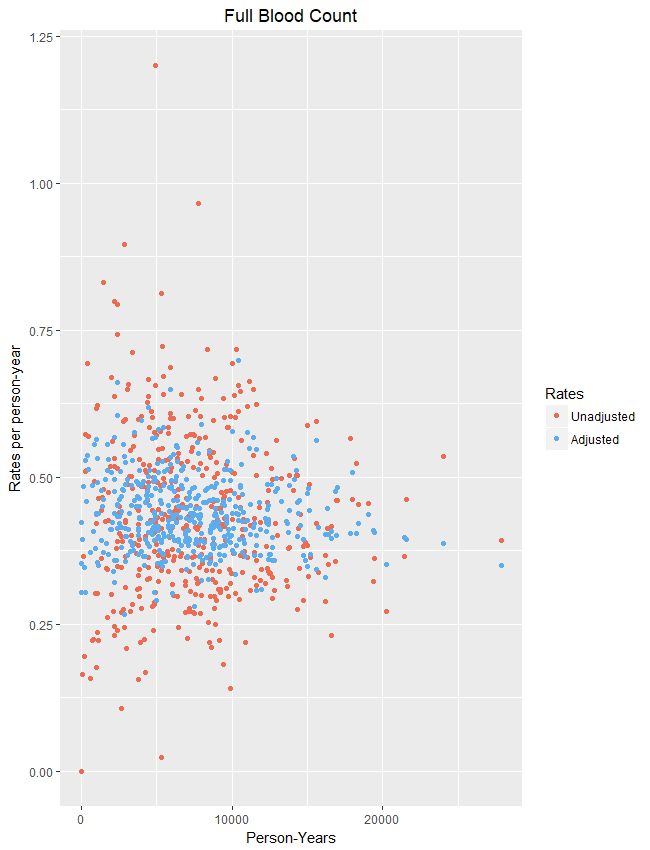

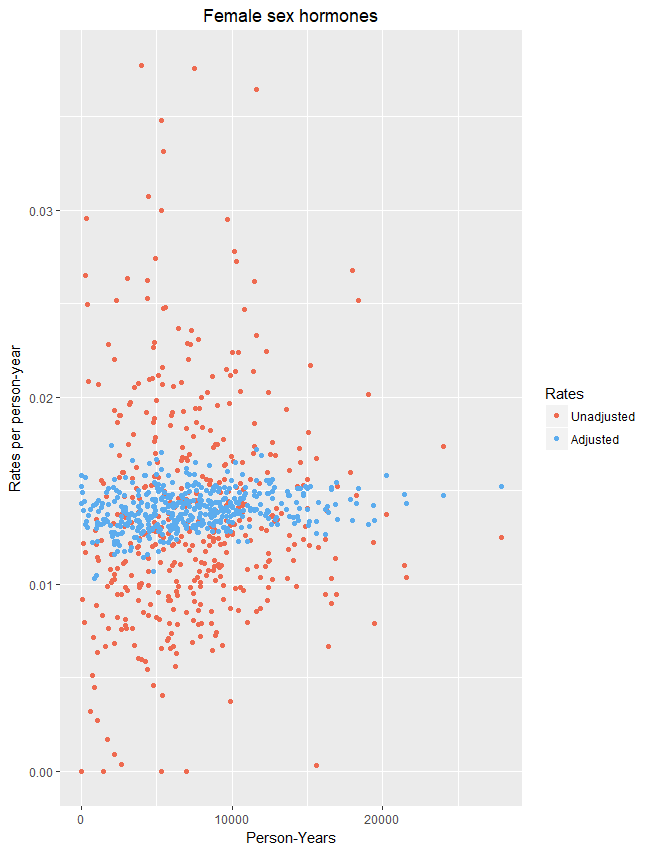

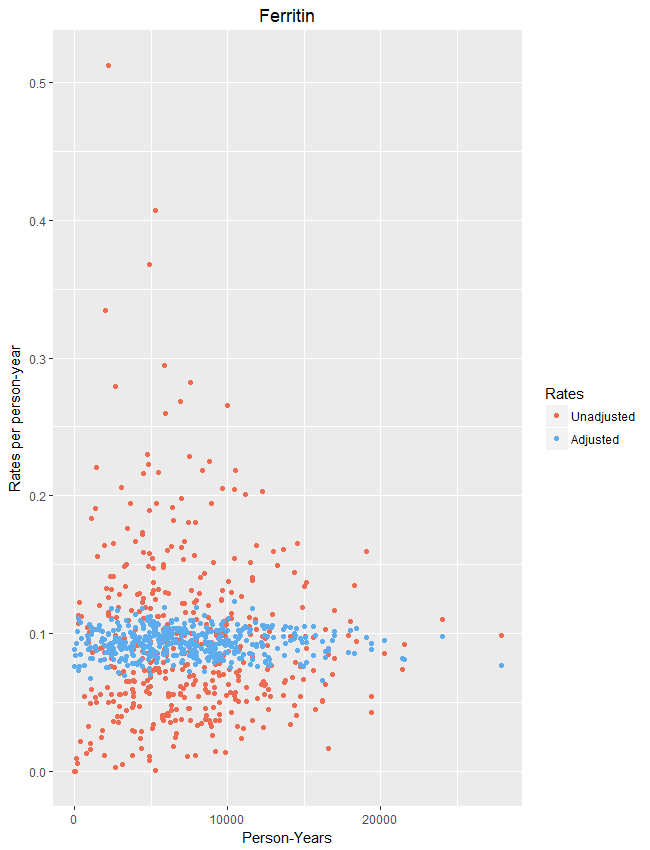

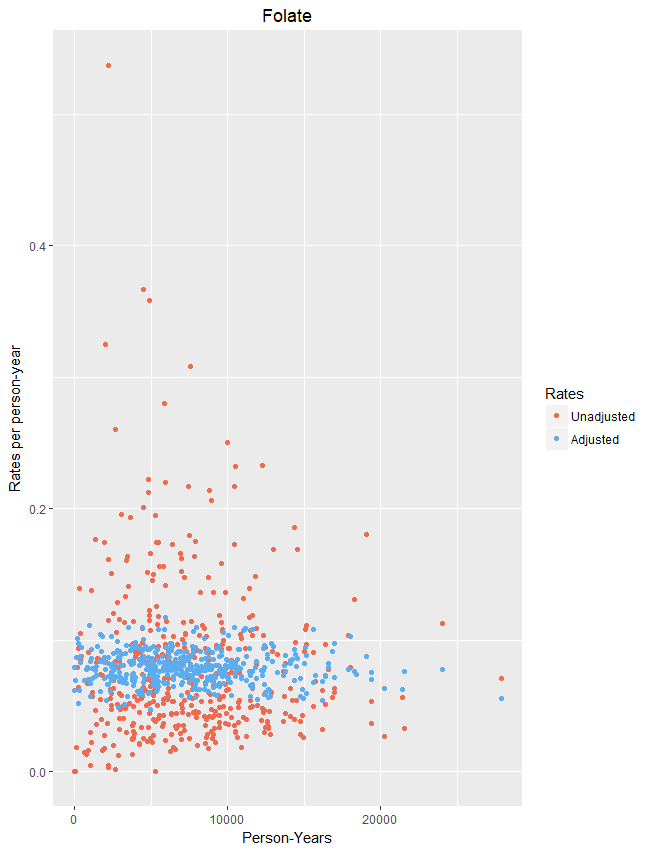

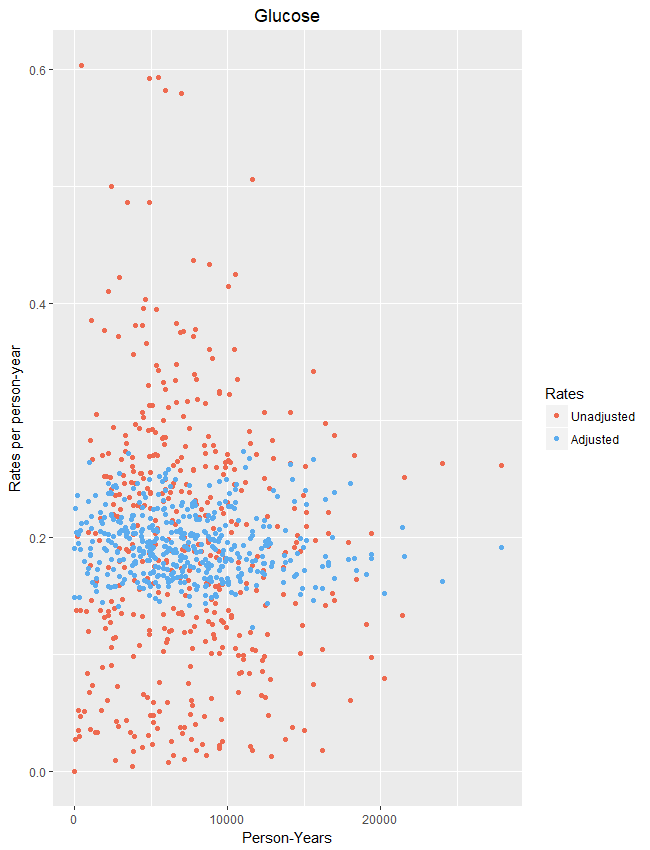

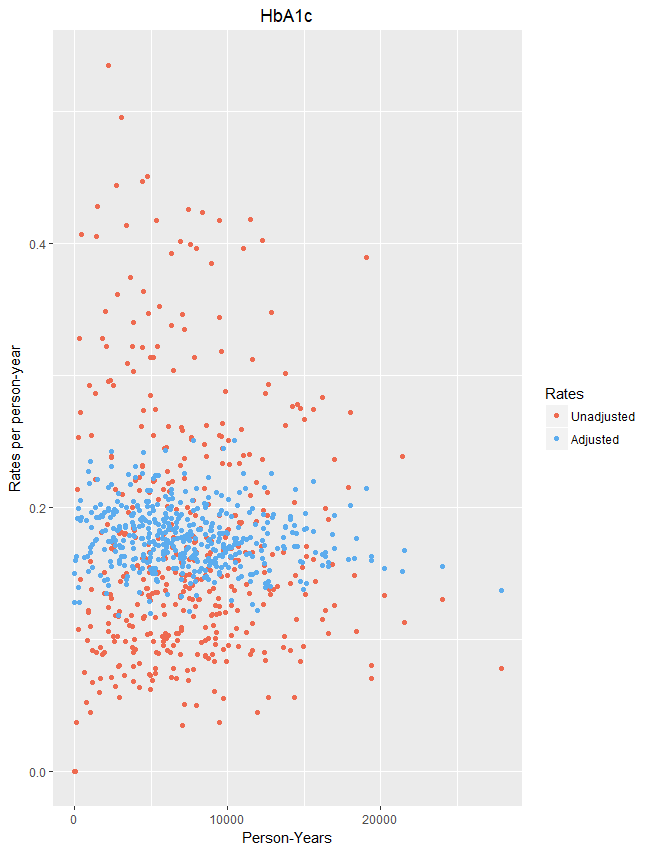

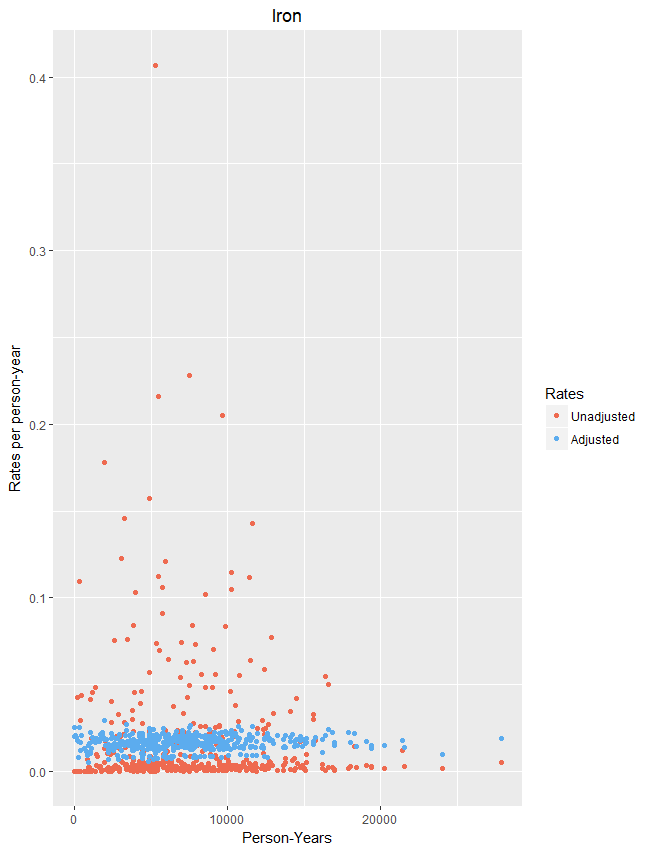

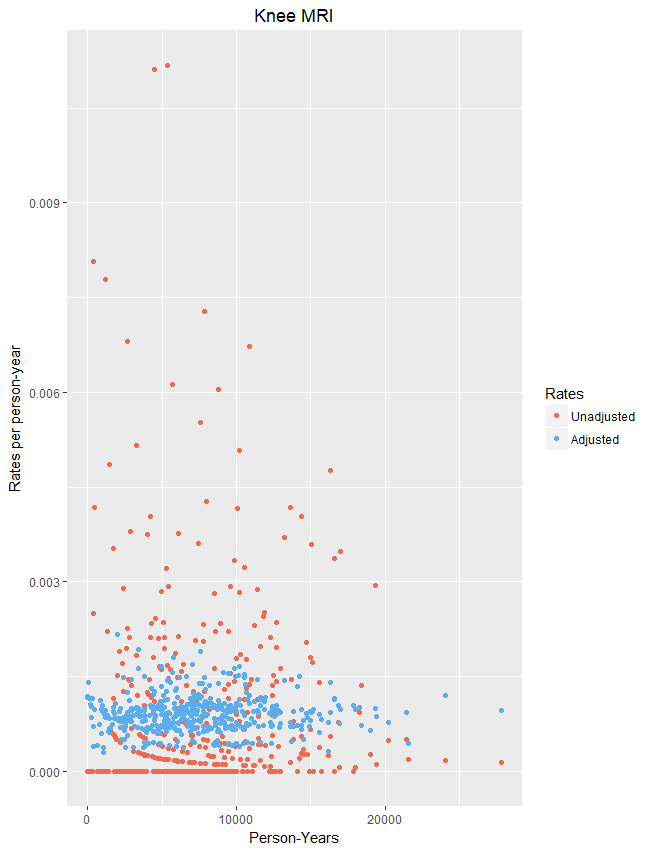

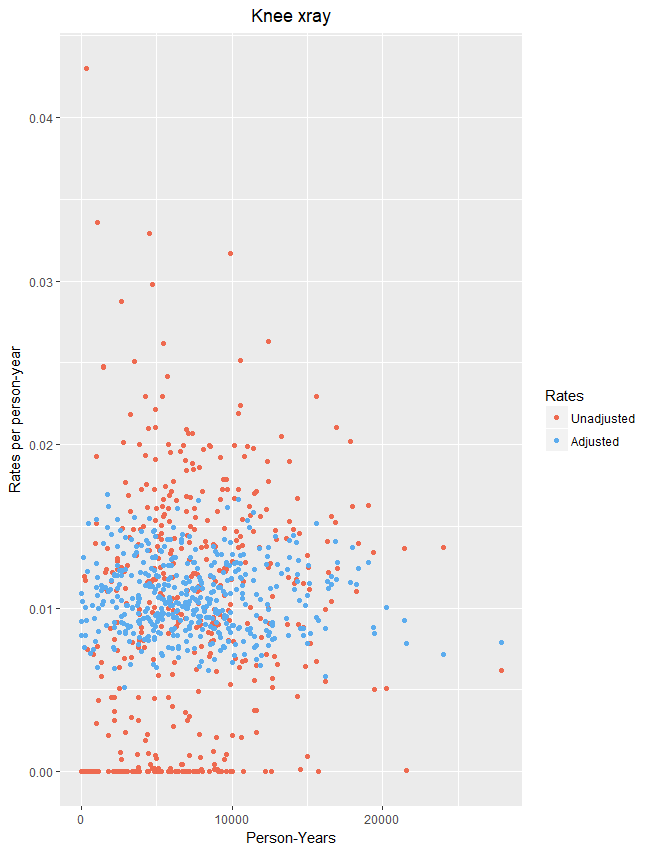

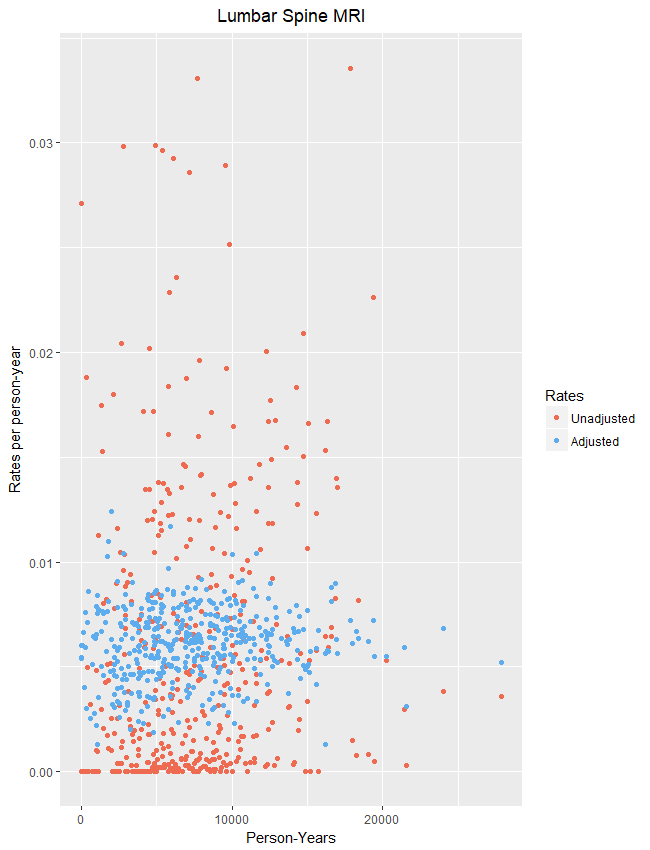

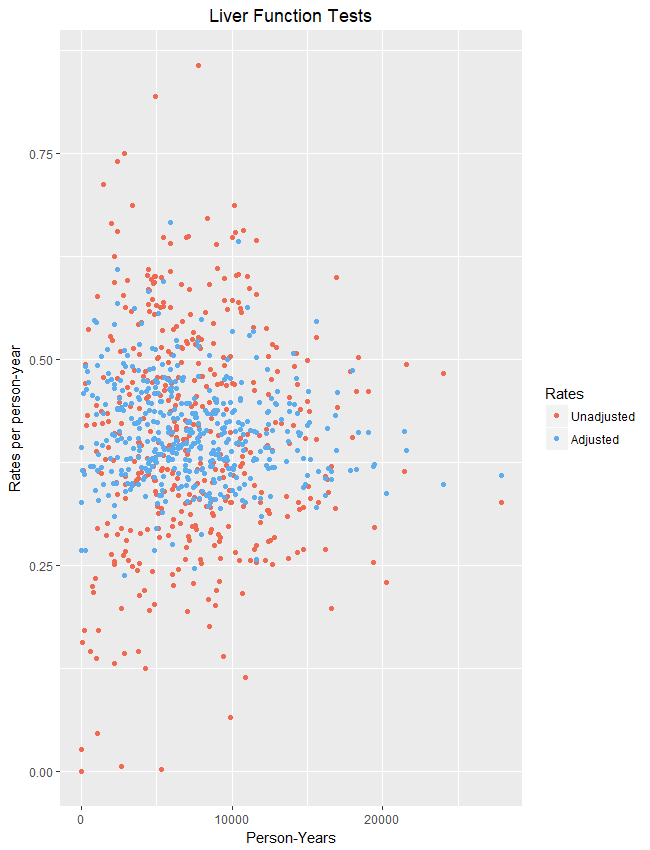

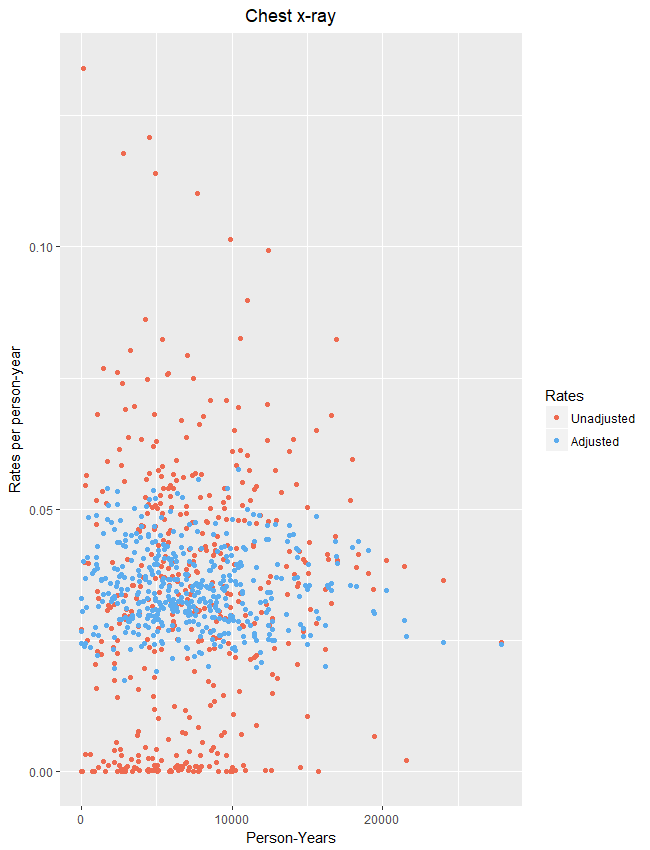

Supplement: Supplementary file 1 — Additional methods and results. (DOCX 725 kb) [file 12916_2018_1217_MOESM1_ESM.docx]
